# Interplay of m^6^A and histone modifications contributes to temozolomide resistance in glioblastoma

**DOI:** 10.1002/ctm2.553

**Published:** 2021-09-15

**Authors:** Fuxi Li, Siyun Chen, Jiaming Yu, Zhuoxing Gao, Zhangyi Sun, Yang Yi, Teng Long, Chuanxia Zhang, Yuzhe Li, Yimin Pan, Chaoying Qin, Wenyong Long, Qing Liu, Wei Zhao

**Affiliations:** ^1^ State Key Laboratory of Ophthalmology, Zhongshan Ophthalmic Center Sun Yat‐sen University Guangzhou China; ^2^ Guangdong Provincial People's Hospital Guangdong Academy of Medical Sciences Guangzhou China; ^3^ Key Laboratory of Stem Cells and Tissue Engineering, Sun Yat‐Sen University, Ministry of Education Guangzhou China; ^4^ Neurosurgery Department, Xiangya Hospital Central South University Changsha China

**Keywords:** glioblastoma, histone modifications, m^6^A, METTL3, TMZ resistance

## Abstract

**Background:**

Despite the development of new treatment protocols for glioblastoma (GBM), temozolomide (TMZ) resistance remains a primary hindrance. Previous studies, including our study, have shown that aberrant N6‐methyladenosine (m^6^A) modification is implicated in GBM pathobiology. However, the roles and precise mechanisms of m^6^A modification in the regulation of TMZ resistance in GBM remain unclear.

**Methods:**

m^6^A individual‐nucleotide‐resolution cross‐linking and immunoprecipitation sequencing (miCLIP‐seq) was performed to identify m^6^A modification of transcripts in TMZ‐resistant and ‐sensitive tumors. To explore the role of METTL3 in TMZ resistance, TMZ‐resistant GBM cells were transfected with *METTL3* shRNA or overexpression lentivirus and then assessed by cell viability, tumor sphere formation, and apoptosis assays. An intracranial GBM xenograft model was developed to verify the effect of METTL3 depletion during TMZ treatment in vivo. ATAC‐seq, ChIP‐qPCR, and dual‐luciferase reporter assays were carried out to verify the role of SOX4/EZH2 in the modulation of METTL3 expression upon TMZ treatment.

**Results:**

We demonstrated that TMZ treatment upregulated the expression of the m^6^A methyltransferase METTL3, thereby increasing m^6^A modification of histone modification‐related gene transcripts. METTL3 is required to maintain the features of GBM stem cells. When combined with TMZ, METTL3 silencing suppressed orthotopic TMZ‐resistant xenograft growth in a cooperative manner. Mechanistically, TMZ induced a SOX4‐mediated increase in chromatin accessibility at the *METTL3* locus by promoting H3K27ac levels and recruiting RNA polymerase II. Moreover, *METTL3* depletion affected the deposition of m^6^A on histone modification‐related gene transcripts, such as EZH2, leading to nonsense‐mediated mRNA decay. We revealed an important role of EZH2 in the regulation of *METTL3* expression, which was via an H3K27me3 modification‐independent manner.

**Conclusions:**

Our findings uncover the fundamental mechanisms underlying the interplay of m^6^A RNA modification and histone modification in TMZ resistance and emphasize the therapeutic potential of targeting the SOX4/EZH2/METTL3 axis in the treatment of TMZ‐resistant GBM.

## Graphical abstract

1. TMZ treatment causes an upregulation of METTL3 expression, thereby increasing m^6^A modification of histone modifiers.

2. TMZ induces SOX4‐mediated increase of chromatin accessibility at *METTL3* locus.

3. METTL3 depletion affects the glioblastoma stem cell features via promoting nonsense‐mediated mRNA decay (NMD) of histone modifiers, such as EZH2.

4. EZH2‐SOX4 complex regulates METTL3 expression in an H3K27me3 independent manner.

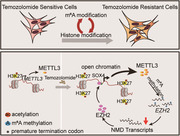

## 1 INTRODUCTION

Glioblastoma (GBM), with a median survival time of less than two years, is considered one of the most common and aggressive primary brain tumors in adults.^1^, ^2^ Standard treatment of newly diagnosed GBM includes surgical resection, radiotherapy, and concomitant chemotherapy. Temozolomide (TMZ) significantly prolongs the median survival period with low toxicity compared to radiotherapy alone, making TMZ the first‐line anti‐GBM drug.^3^, ^4^ Unfortunately, at least half of GBM patients do not respond to TMZ. To make things even worse, most patients who have good responses eventually develop resistance to TMZ during the treatment. Although great efforts have been made to determine the possible causes, the complete mechanism of TMZ resistance remains unclear.

The most well‐known mechanism of TMZ resistance is O6‐methylguanine‐DNA methyltransferase (MGMT) overexpression, which mediates TMZ resistance by repairing the main cytotoxic lesions.^5^ Mismatch repair (MMR) defects are also common mechanisms underlying acquired resistance to TMZ.^6^ Increasing evidence suggests that MGMT overexpression and MMR deficiency may not be the only molecular mechanisms underlying TMZ resistance in GBM patients as histone modification factors, microRNAs, and long noncoding RNAs may also be involved. For example, a recent study demonstrated that the EZH2/ATRX complex contributes to TMZ resistance by regulating the FADD/PARP1 axis.^7^ Using lncRNA microarray screening, Wu et al found an unreported lncRNA, lnc‐TALC, regulating TMZ resistance by competitively binding miR‐20b‐3p and facilitating c‐Met expression.^8^ These studies indicate that epigenetic regulation plays a critical role in TMZ resistance.

N6‐methyladenosine (m^6^A) is the most prevalent epigenetic modification of mRNA in eukaryotic cells.^9^, ^10^ m^6^A regulates the expression of a series of genes by modulating every stage of mRNA metabolism, including pre‐mRNA splicing,^11^ 3′‐end processing,^12^ nuclear export,^13^ mRNA translation,^14^, ^15^ and mRNA decay.^16^, ^17^ Our previous work indicated that, in GBM, m^6^A regulates nonsense‐mediated mRNA decay (NMD),^18^ which is the most conserved mRNA quality control mechanism for the removal of mRNAs harboring premature stop codons (PTCs) or short upstream open reading frames. Moreover, m^6^A modification is dynamic and reversible, and accomplished by the cooperation of m^6^A methyltransferases (METTL3, METTL14, and WTAP),^19^ demethylases (FTO and ALKBH5),^20^, ^21^ and “readers” (YTHDF1‐3 and YTHDC1‐2).^22^ Recent evidence, including our work, suggests a relationship between m^6^A modification and cancer progression.^18^, ^23^, ^24^, ^25^ However, the role of m^6^A in TMZ resistance in GBM is undetermined.

Previous studies have suggested that m^6^A modification is associated with drug resistance in many types of tumors, but the role of m^6^A is still controversial.^26^, ^27^ The reasons for contradictory conclusions may be because of different cancer types, intratumor heterogeneity, and compensatory epigenetic changes in cancer cells. Furthermore, we believe that dynamic changes in m^6^A modification at specific gene loci are more important for drug resistance than changes in the total number of m^6^A modifications.^28^, ^29^, ^30^ To this end, we performed m^6^A individual‐nucleotide‐resolution crosslinking and immunoprecipitation sequencing (miCLIP‐seq) to map the m^6^A locations with single‐nucleotide resolution in TMZ‐resistant and ‐sensitive clinical GBM samples. We sought to assess the biological function of m^6^A in TMZ resistance in GBM patients and investigate the underlying epigenetic mechanism by determining the critical targets of m^6^A. Our results indicated that TMZ treatment induced the expression of the m^6^A methyltransferase, METTL3, in GBM cells via the SOX4/EZH2/H3K27ac cascade, thereby promoting m^6^A modification of the transcripts of histone modification factors. Collectively, our findings reveal crucial crosstalk between m^6^A and histone modifications in TMZ resistance and emphasize the therapeutic potential of targeting the SOX4/EZH2/METTL3 axis for the treatment of GBM.

## 2 MATERIALS AND METHODS

### 2.1 Glioma specimen collection

GBM surgical specimens were collected at Xiangya Hospital of Central South University, in accordance with institution‐approved protocols. Written informed consent was obtained from each study participant after a thorough explanation of the procedure and its risk, in compliance with the Declaration of Helsinki.

The DNA methylation status of MGMT promoter was used as a surrogate marker of intrinsic resistance to TMZ for these clinical tumors.

### 2.2 Cell lines and primary GBM cell cultures

The human GBM cell lines U251 and U87MG were provided by Dr. Jun Cui (Sun Yat‐sen University) and grown in Gibco^®^ Dulbecco's Modified Eagle's medium (DMEM) containing 10% fetal bovine serum (FBS, Gibco, USA) at 37°C in a humidified atmosphere containing 5% CO_2_. Cells were tested for mycoplasma contamination every 2 weeks.

Primary GBM specimens were minced in sterile phosphate‐buffered saline (PBS) on ice and then pressed through 70 μm cell strainers (Falcon, USA) to generate a single‐cell suspension. Next, the primary GBM cells were collected and cultured in ultralow attachment 24‐well plates with stem cell medium (DMEM/F12 supplemented with 15% FBS [Gibco], 20 ng/mL EGF [CantonBIO, Guangzhou, China], 1 × B27 [Invitrogen, USA], and 20 ng/mL bFGF [CantonBIO]). All patient‐related studies were approved by the Research Ethics Board at Xiangya Hospital of Central South University.

### 2.3 Establishment of TMZ‐resistant cells

TMZ‐resistant U87MG_TMZ_R cells were generated using U87MG cells. Briefly, U87MG cells were cultured in 12‐well plates with 50 μM TMZ (T1178; TargetMol, USA) and the cell culture medium for 1 month. The cells were then exposed to increasing concentrations of TMZ (50, 100, 150, 200, 250, and 300 μM) for 6 months. The induced U87MG_TMZ_R cells were maintained in 300 μM TMZ medium.

### 2.4 Cell viability and TMZ treatment

CellTiter‐Glo Luminescent Cell Viability Assay (Promega, Madison, WI, USA) was used to determine GBM cell viability, according to the manufacturer's instructions. Cells were seeded in 96‐well plates in 100 μL of Gibco^®^ DMEM containing 10% FBS at a density of 2 × 10^3^ cells per well. The cells were incubated at 37°C in a humidified 5% CO_2_ atmosphere with different concentrations of TMZ (TargetMol), and the culture medium was discarded at 72 h. Cell lysis was induced by adding 40 μL of CTG solution to each well and incubating for 20 min at 37°C with rotation followed by recording luminescence.

### 2.5 Tumor sphere formation assays

Cells were seeded in ultralow attachment 6‐well plates (Corning, USA) at a density of 5 × 10^3^ cells per well. The cells were cultured in DMEM/F12 supplemented with 1 × B27, 20 ng/mL EGF, 20 ng/mL bFGF, and dimethyl sulphoxide (DMSO) or 800 μM TMZ. Tumor spheres were harvested on the seventh day after cell seeding.

### 2.6 Intracranial GBM xenograft model

Six‐week‐old female BALB/c nude mice were purchased from the Model Animal Research Center of Nanjing University and housed in individually ventilated micro‐isolator cages under a 12 h dark/light cycle. Nude mice were divided into four groups of six mice each. After deep anesthesia, a 27‐gauge needle was used to drill a burrhole into the skull 0.5 mm anterior and 2 mm lateral to the bregma. A 10 μL gas‐tight syringe (Hamilton) was then used to inject 10 μL of the U87 MG‐TMZ_R‐luc cell suspension in the striatum at a depth of 3 mm from the dural surface. One week after the injection of the tumor cells, 40 mg/kg/day of TMZ in saline was administered for over 2 weeks by intraperitoneal injection. During TMZ treatment, a Xenogen IVIS Spectrum system was used to monitor the tumor growth. At the end point, brain tissues were dissected from the mice models and measured the length (a) and width (b) of the tumors. Tumor volume was calculated by the formula *V* = *ab* ^2^/2.

### 2.7 Identification of NMD target transcripts

Genomic alignments were constructed by splitting the human *EZH2*, *KMT5A*, *KAT2A*, and *SFPQ* gene loci into individual exon and intron sequences and aligning each pair of orthologous sequences using the global alignment program of FASTA v2.1. The ESEfinder web server was used to search for *EZH2*, *KMT5A*, *KAT2A*, and *SFPQ* genes containing a PTC exon for sites scoring higher than the default thresholds.

### 2.8 miCLIP‐seq and analysis

The miCLIP‐seq assay was performed as previously reported^31^ with some modifications. In brief, Trizol solution (Life Technologies, USA) was used to extract total RNA from tissue samples and cell lines, followed by mRNA purification using the Dynabeads mRNA Purification Kit (Invitrogen, USA). After digesting genomic DNA with RNase‐free DNase I (Thermo Scientific, USA), the resulting mRNA was purified using the RNA Clean & Concentrator kit (Zymo, Irvine, CA, USA) and resuspended at a concentration of 1 μg/μL. Purified mRNAs (10 μg) were fragmented to an average size of 30‐130 nucleotides using the fragmentation reagent (Life Technologies, USA), mixed in 480 μL immunoprecipitation buffer (50 mM Tris‐HCl, pH 7.4, 150 mM NaCl, 0.5% NP‐40) with 10 μL of 1 mg/mL anti‐m^6^A antibodies (Abcam, ab151230), and incubated at 4°C with rotation for 1.5‐2 h. The solution was then irradiated three times with 254 nm light at a dose of 150 mJ/cm^2^ in a clear 12‐well tissue culture plate on ice. The sample was further incubated with protein A/G magnetic beads (Thermo Scientific, 88802). After washing procedures and on‐bead linker ligation, the magnetic beads were resuspended in NuPage LDS sample buffer (Invitrogen, USA) and incubated at 70°C with constant shaking for 15 min. The RNA‐antibody complex was purified using sodium dodecyl sulphate‐polyacrylamide gel electrophoresis (SDS‐PAGE) (NuPage Novex 4%‐12% Bis‐Tris protein gel) and transferred to polyvinylidene fluoride (PVDF) membranes. Subsequently, the part of the membrane that contained the whole protein lane was excised. RNA bound to the membranes was eluted using proteinase K, isolated by acidic phenol/chloroform/isoamyl alcohol extraction, and precipitated in ethanol. The purified RNA fragments were reverse transcribed using SuperScript III reverse transcriptase (Life Technologies, USA). We used CircLigase II (Epicenter, Madison, WI, USA) and FastDigest *BamHI* (NEB, Ipswich, MA, USA) to circularize and relinearize the purified 85‐200 nucleotide first‐strand cDNA, respectively. Sequencing libraries were prepared using AccuPrime SuperMix I (Invitrogen, USA), and sequencing was performed on an Illumina HiSeq 2500. Raw sequencing reads were processed as previously described,^32^ calling m^6^A sites as crosslinking‐induced mutation sites (CIMS). The CTK package (https://zhanglab.c2b2.columbia.edu/index.php/CTK_Documentation) was used to identify base substitution events in a DRACH consensus sequence, and C to T transitions were used to identify m^6^A sites and A to T transitions were used to identify m^6^Am sites. After extracting the A to T transitions for each mutation position, the CIMS.pl program was used to determine the coverage of the unique tag (*k*) and mutations (*m*). We retained the positions within 1% ≤ *m*/*k* ≤ 50% for mining and analyzing the Metagene plot of miCLIP and genomic distribution of CIMS.

### 2.9 ATAC‐seq and analysis

Chromatin preparation Nuclei were prepared from U87MG_TMZ_R cells (2 × 10^4^). TruePrepTM DNA Library Prep Kit V2 for Illumina (TD501, Vazyme, Nanjing, China) and TruePrepTM Index Kit V2 for Illumina^®^ (TD202, Vazyme) were used to prepare DNA libraries according to the manufacturer's instructions. Libraries were then quantified by qPCR and sequenced using 150 bp paired‐end reads and dual‐index sequencing on a Nova‐Seq instrument.

Raw reads were adaptor‐trimmed using Trim Galore (v0.6.4) and aligned to the genome using Bowtie2 (v2.2.3) with the “very‐sensitive” option. Peaks were called using the MACS2 software (v2.2.7.1) with the option *P* < 0.005 to retain significant peaks, while default parameters were used for other options.

### 2.10 Bisulfite sequencing PCR

The genomic DNA of U87‐MG cells was extracted using a DNA extraction kit (TIANGEN, Beijing, China). Bisulfite treatment was conducted using the EpiArt DNA Methylation Bisulfite Kit (Vazyme) according to the manufacturer's protocol. Forward (5′‐TAGTATTTTGGGAGGTTAAGGAGG‐3′) and reverse (5′‐AAAAACAACACCATATAATACAATTT‐3′) primers were used to amplify the region of interest (ROI), and the PCR products were ligated into the pUCmT vector (Sangon Biotech, Shanghai, China) for cloning and sequencing. Analysis of the degree of DNA methylation was performed using the DNAMAN analysis software.

### 2.11 Statistical analysis

All statistical analyses were performed using GraphPad Prism version 8.0 (GraphPad Software, USA). Survival curves were plotted using the Kaplan‐Meier method. Data are presented as mean ± SD of three independent experiments. Comparisons between two groups were performed using a two‐sided Student's *t*‐test. For all tests, a *P* value less than 0.05 was considered statistically significant and marked as “*”; a *P* value less than 0.01 was marked as “**”; and a *P* value less than 0.001 was marked as “***.”

## 3 RESULTS

### 3.1 The m^6^A transcriptome‐wide profile in the TMZ‐resistant GBM tumor

**FIGURE 1:**
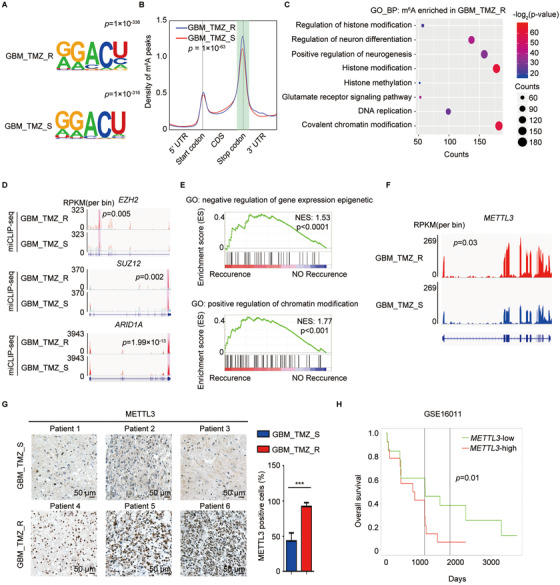
Difference in m^6^A methylome between TMZ‐resistant and ‐sensitive GBM patient samples. (A) Motif analysis of m^6^A modification peaks in TMZ‐resistant and ‐sensitive GBM miCLIP‐seq data. (B) Distribution of m^6^A modification peaks across all mRNAs in TMZ‐resistant and ‐sensitive GBM. GBM_TMZ_R exhibited higher amount of m^6^A levels around stop codon (shadow) compared with GBM_TMZ_S (Student's *t*‐test). (C) Gene Ontology (GO) analyses of genes with increased m^6^A modifications in TMZ‐resistant GBM sample. (D) m^6^A modification status of histone modification‐related genes *EZH2*, *SUZ12*, and *ARID1A* in TMZ‐resistant and ‐sensitive GBM samples. The *y*‐axis shows the nomalized RPKM (per bin, bin = 25 bp) value. Exomepeak R package was used for statistical comparison. (E) Gene Set Enrichment Analysis (GSEA) plots show the selected GO gene sets enriched in recurrent patients after TMZ therapy. (F) Integrative genomics viewer (IGV) plots of RNA‐seq peaks at *METTL3* mRNA. The *y*‐axis shows the normalized RPKM (per bin, bin = 25 bp) value. DESeq2 was used for statistical comparison. (G) IHC staining of METTL3 in TMZ‐resistant (*n* = 3) and TMZ‐sensitive (*n* = 3) GBM tumors. The statistical results show the proportion of METTL3‐positive cells in each group. (H) Association between *METTL3* expression and overall survival of the GBM patients from GSE16011 datasets was analyzed by Kaplan‐Meier analysis. *P < 0.05; **P < 0.01; and ***P < 0.001; n.s., no significant difference, compared to control (Student's *t*‐test). All the results were obtained from three independent experiments. Values are presented as mean ± SD. GBM_TMZ_R, TMZ‐resistant GBM patient; GBM_TMZ_S, TMZ‐sensitive GBM patient

To obtain a transcriptome‐wide m^6^A map in TMZ‐resistant GBM, we first detected m^6^A modification in two GBM tumors from patients with or without TMZ resistance by miCLIP‐seq. The motifs of m^6^A peaks in both samples were found to be consistent with those of the consensus sequence of RRACH (Figure 1A). The distribution pattern analyses suggested that the peaks were highly enriched in the 3′ UTR and coding sequence (CDS) regions in both resistant and sensitive tumor tissues (Figure S1A). TMZ‐resistant GBM exhibited greater total m^6^A levels (Figure S1B) and increased m^6^A signal around the stop codon (Figure 1B) compared to TMZ‐sensitive GBM. We further confirmed the preferential locations of m^6^A peaks in these two samples. There were 4772 genes containing increased number of or new m^6^A peaks in the TMZ‐resistant sample compared to the TMZ‐sensitive sample (Figure S1C). The 4772 genes with increased m^6^A modification in TMZ‐resistant samples were used to perform gene ontology (GO) analysis. The significant genes were related to the regulation of histone modification, histone methylation, and covalent chromatin modification (Figure 1C). Indeed, several histone modification‐related genes, including *EZH2*, *SUZ12*, *ARID1A*, *ARID1B*, *SMARCA2*, and *SUV39H1*, showed increased m^6^A modification in TMZ‐resistant samples compared to TMZ‐sensitive samples (Figures 1D and S1D). Notably, recurrent patients had considerably higher expression of genes related to epigenetic regulation compared to nonrecurrent patients (Figure 1E). Taken together, there was a tendency for a positive correlation between m^6^A methylation and histone modification‐related gene expression.

The high m^6^A levels in TMZ‐resistant GBM patients are most likely due to abnormal expression of m^6^A modulators; thus, we detected the expression of m^6^A modulators in chemotherapy‐resistant patients by analyzing the RNA‐seq data. *METTL3* expression was significantly elevated in TMZ‐resistant GBM samples (Figure 1F). Few changes in mRNA expression were observed in other m^6^A modulators (Figure S1E). Moreover, IHC staining revealed that TMZ‐resistant GBM tissues (*n* = 3) exhibited significantly higher expression of METTL3 compared to TMZ‐sensitive samples (*n* = 3) (Figure 1G). Furthermore, patients with high *METTL3* expression had a poor prognosis during TMZ therapy (Figure 1H). Collectively, METTL3 expression is upregulated and acts as a crucial component of the m^6^A methyltransferase complex, promoting m^6^A levels in TMZ‐resistant GBM cells.

### 3.2 METTL3 expression is regulated by alterations in chromatin accessibility in GBM cells

**FIGURE 2:**
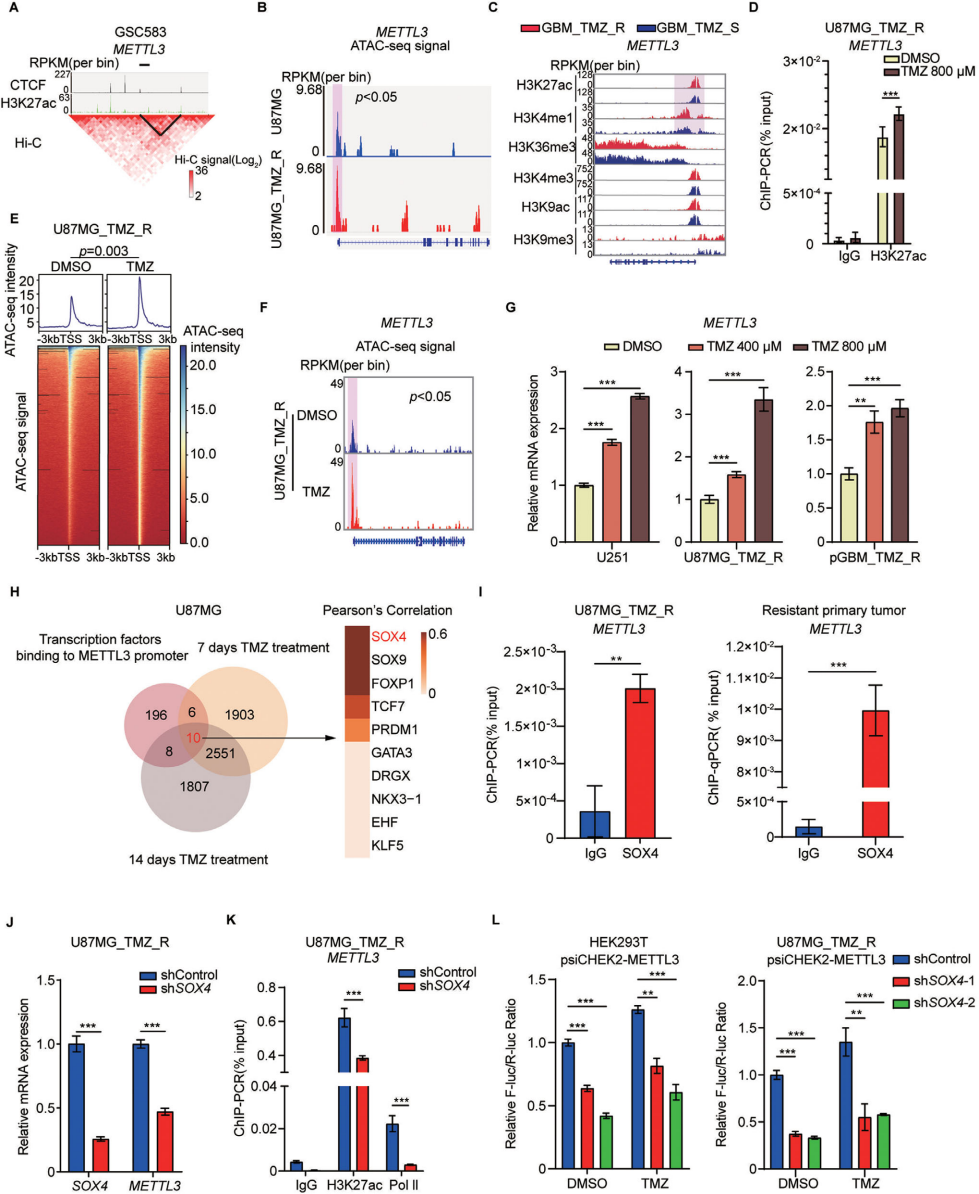
SOX4 participates in TMZ‐induced enhancement of transcription of *METTL3*. (A) Comprehensive analysis of the *METTL3* locus in the GSC583 sample (datasets from http://promoter.bx.psu.edu/hi‐c/). METTL3 locus displayed highly accessible and active in gioblastoma, marked by higher enrichment of H3K27ac, which are proximal to the boundary of topological domain. (B) IGV plots of ATAC‐seq peaks at the *METTL3* locus in TMZ‐sensitive U87MG cells and TMZ‐resistant U87MG cells (U87MG_TMZ_R). The *y*‐axis shows the normalized RPKM (per bin, bin = 25 bp) value. ATAC‐seq signal around TSS of METTL3 (shadow) was compared by MACS2. (C) Integrated analysis of histone modifications at the *METTL3* locus (datasets from GSE113816). The *y*‐axis shows the normalized RPKM (per bin, bin = 25 bp) value. (D) ChIP‐qPCR analysis of H3K27ac enrichment at the *METTL3* promoter region in U87MG_TMZ_R cells treated with DMSO or TMZ. (E) Heatmap showing the ATAC‐seq signal at transcription start sites (TSSs) ± 3 kb regions for all genes in U87MG_TMZ_R cells treated with DMSO or 800 μM TMZ. ATAC‐seq signal at TSSs in METTL3 KD and control U87MG_TMZ_R cell was compared by Student's *t*‐test. (F) IGV plots of ATAC‐seq peaks at the *METTL3* locus in U87MG_TMZ_R cells treated with DMSO or 800 μM TMZ. The *y*‐axis shows the normalized RPKM (per bin, bin = 25 bp) value. ATAC‐seq signal around TSS of METTL3 (shadow) was compared by MACS2. (G) qRT‐PCR analysis of *METTL3* expression in U251, U87MG_TMZ_R, and pGBM_TMZ_R cells treated with DMSO or different concentrations of TMZ for 72 h. (H) Venn diagram showing the shared 10 potential TFs binding to *METTL3* promoters and upregulated in U87MG cells genes after 7 or 14 days of TMZ treatment. Pearson's correlation coefficients of these 10 TFs are shown on the right. (I) ChIP‐qPCR analysis of SOX4 enrichment at the *METTL3* promoter region in U87MG_TMZ_R cells and TMZ‐resistant GBM. (J) mRNA expression of *SOX4* and *METTL3* in *SOX4* KD (pooled *SOX4* shRNAs) or control U87MG_TMZ_R cells. (K) ChIP‐qPCR analysis of H3K27ac and RNA Pol II enrichment at the *METTL3* promoter region in *SOX4* KD (pooled *SOX4* shRNAs) or control U87MG_TMZ_R cells. (L) Dual‐luciferase reporter assay for the effects of *SOX4* KD on the luciferase activity of the *METTL3* promoter (–3000 bp‐0 bp) in HEK293T and U87MG_TMZ_R cells. *P < 0.05; **P < 0.01; and ***P < 0.001, compared to control (Student's *t*‐test). All the results were obtained from three independent experiments. Values are presented as mean ± SD

We speculated that METTL3 expression might be affected by DNA methylation changes during TMZ treatment. However, there was no significant difference in DNA methylation at the promoter regions of the *METTL3* locus between the TMZ‐treated and control groups (Figure S2A). We then analyzed publicly available datasets and found *METTL3* locus showed high accessibility and activity in GBM cells, which was marked by high enrichment of H3K27ac near the boundary of topological domain (Figure 2A). To investigate whether TMZ resistance primarily affects chromatin regulation to increase METTL3 expression, we established a TMZ‐resistant U87MG cell line (U87MG_TMZ_R) and a primary TMZ‐resistant GBM cell line (pGBM_TMZ_R) from a TMZ‐resistant GBM tumor (Figure S2B). There was no significant difference in the DNA methylation of *MGMT* promoter between U87MG_TMZ_R cells and parental U87MG cells (Figure S2C). However, the expression of mismatch repair gene *MSH6* in U87MG_TMZ_R cells was slightly lower than that in U87MG cells (Figure S2D). ATAC‐seq identified regions of open chromatin that were correlated with TMZ resistance (Figure S2E). Additionally, increased peaks in TMZ‐resistant cells were found in the promoter region of *METTL3* (Figure 2B). The promoter regions of *METTL3* had remarkably higher levels of the active histone markers H3K27ac and H3K4me1 in TMZ‐resistant cells than in TMZ‐sensitive tumors (Figure 2C). Likewise, H3K27ac modification on *METTL3* promoter in U87MG_TMZ_R cells was higher than that in U87MG cells (Figure S2F). Furthermore, the enrichment of H3K27ac at the *METTL3* promoter region significantly increased upon TMZ treatment (Figure 2D).

Based on the above results, we proposed that TMZ treatment interferes with the chromatin state. Indeed, ATAC‐seq analyses showed that TMZ treatment resulted in a global increase in chromatin accessibility (Figure 2E). Notably, ATAC‐seq peaks near the *METTL3* promoter in TMZ‐treated cells were higher than those in untreated cells (Figure 2F). TMZ treatment increased the expression of METTL3 in TMZ‐resistant and ‐sensitive cells (Figures 2G and S2G). Consistently, the expression of *METTL3* was higher in TMZ‐resistant U87MG cells than in original U87MG cells (Figure S2H). Moreover, treatment with the BRD4 inhibitor JQ1 reversed the TMZ‐induced increase in METTL3 expression (Figure S2I).

To investigate how TMZ can activate *METTL3* transcription, we used the JASPAR website (http://jaspar.genereg.net/) and predicted 220 transcription factors (TFs) binding to the *METTL3* promoter region (Figure 2H). Additionally, RNA‐seq assay showed consistent differentially expressed genes (2561 genes) in U87MG cells upon TMZ treatment for 7 and 14 days (Figure 2H). The 10 overlapping TFs were then analyzed using the Pearson correlation of *METTL3* expression (Figure 2H). SOX4 was identified as the most likely candidate TF modulating *METTL3* expression upon TMZ treatment (Figures 2H and S2J). Indeed, our ChIP‐qPCR results confirmed that the *METTL3* locus was strongly bound by SOX4 in TMZ‐resistant cells and GBM tumors (Figure 2I). We found that knockdown (KD) of *SOX4* significantly downregulated *METTL3* expression (Figure 2J) and decreased the level of H3K27ac and RNA polymerase II (Pol II) at the *METTL3* promoter region (Figure 2K). Importantly, KD of *SOX4* rescued the increase in METTL3 reporter activity induced by TMZ treatment (Figure 2L). We also found that *SOX4* expression was higher in TMZ‐resistant GBM tumors than in TMZ‐sensitive GBM tumors (Figure S2K).

### 3.3 METTL3 is essential for maintaining TMZ resistance in GBM cells

**FIGURE 3:**
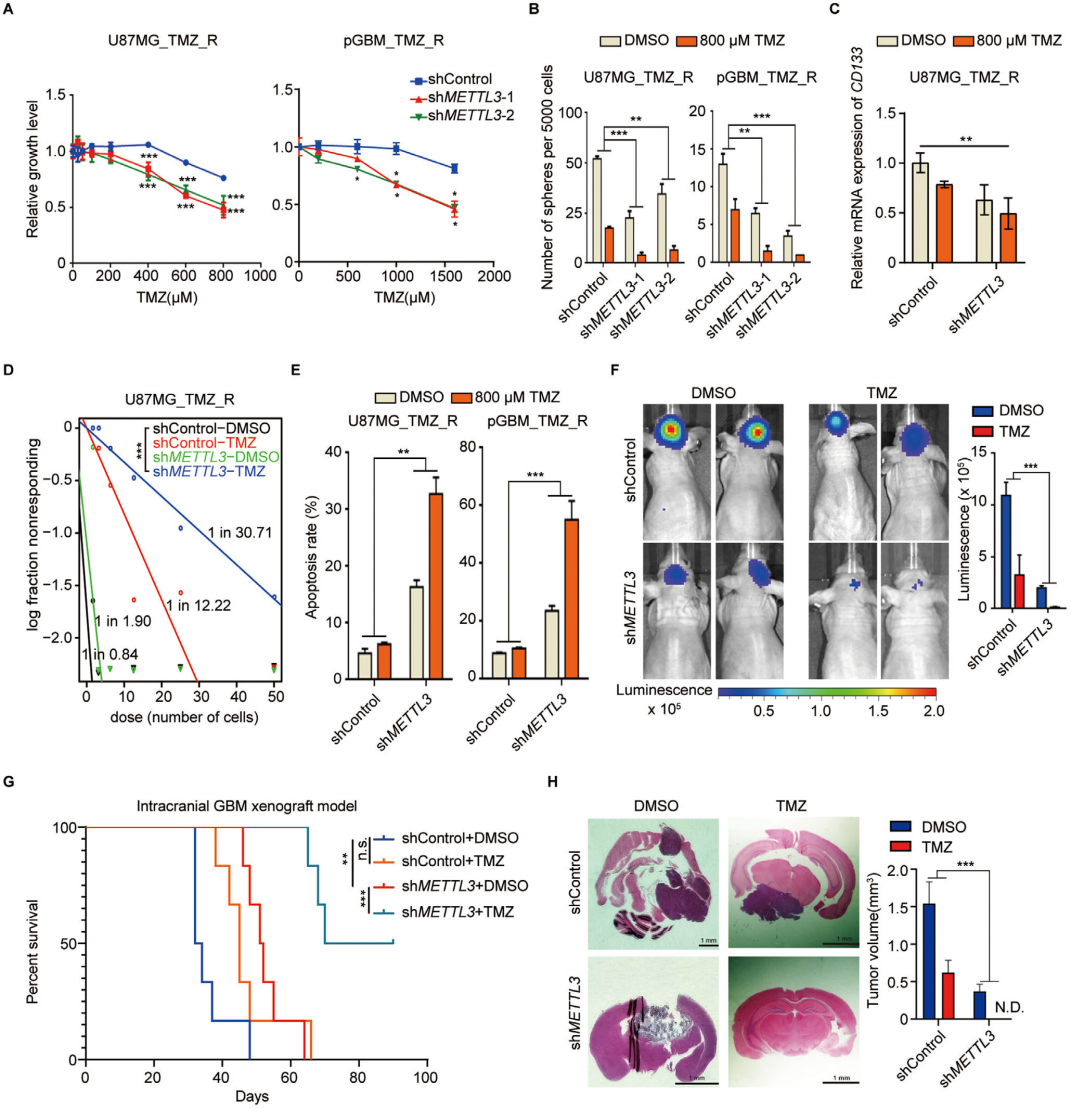
METTL3 inhibition enhances sensitivity of TMZ‐resistant GBM cells to TMZ. (A) Cell viability assays of U87MG_TMZ_R and pGBM_TMZ_R cells transduced with sh*METTL3* and treated with different concentrations of TMZ were performed using CellTiter‐Glo. U87MG_TMZ_R, TMZ‐resistant U87MG cells; pGBM_TMZ_R, TMZ‐resistant primary GBM cells. (B) Sphere formation assay of U87MG_TMZ_R and pGBM_TMZ_R cells after *METTL3* silencing and TMZ treatment compared with the control (two‐way ANOVA). The number of spheres formed was counted on day seven. (C) Expression of *CD133* in U87MG_TMZ_R‐derived spheres (7 days) after *METTL3* silencing and TMZ treatment compared with the control. (D) Limiting dilution assay (LDA) of U87MG_TMZ_R‐derived sphere cells after *METTL3* silencing (pooled *METTL3* shRNAs) and TMZ treatment compared with the control. (E) Proportion of apoptotic cells in *METTL3* KD (pooled *METTL3* shRNAs), control U87MG_TMZ_R, and pGBM_TMZ_R cells following TMZ treatment for 72 h (two‐way ANOVA). (F) Representative images of brain tumors in mice intracranially injected with sh*METTL3* (pooled *METTL3* shRNAs)‐treated or control TMZ‐resistant U87MG cells and treated with TMZ (40 mg/kg/day) (two‐way ANOVA). The scale bar of bioluminescence intensity is shown at the bottom. (G) Kaplan‐Meier survival curve for the four different treatment groups. (H) Representative images of H&E‐stained sections of the brain tissue of mice at 4 weeks after the intracranial injection of sh*METTL3*‐treated (pooled *METTL3* shRNAs) or control TMZ‐resistant U87MG cells and treated with TMZ (40 mg/kg/day). *P < 0.05; **P < 0.01; and ***P < 0.001, compared to control (Student's *t*‐test and two‐way ANOVA). All the results were obtained from three independent experiments. Values are presented as mean ± SD

To determine the functional role of METTL3 in TMZ resistance, we established *METTL3* KD U87MG_TMZ_R cells and pGBM_TMZ_R cells using two independent shRNAs (Figure S3A). *METTL3* KD reduced total m^6^A modification (Figure S3B) and restored the sensitivity of TMZ‐resistant GBM cells to TMZ treatment (Figures 3A and S3C), while ectopic expression of *METTL3* (Figure S3D) enhanced resistance to TMZ (Figure S3E). Overexpression of *METTL3* increased the DNA damage response (DDR) to TMZ treatment in TMZ‐resistant cells (Figure S3F). In addition, *METTL3* KD significantly decreased sphere formation capacity (Figure 3B), GSC marker expression (Figure 3C), and self‐renewal (Figure 3D) in TMZ‐resistant cells. Moreover, the apoptosis assay results indicated that TMZ treatment remarkably increased apoptosis of *METTL3* KD cells, but had no effect on control TMZ‐resistant cells (Figure 3E).

From in vivo bioluminescence imaging, we observed that *METTL3* KD caused a significant reduction in tumor size and promoted TMZ sensitivity with respect to the control cells (Figure 3F). Mice with shMETTL3‐treated cells showed favorable survival and greater benefit from TMZ therapy compared to control mice (Figure 3G). Hematoxylin and eosin stain (H&E) staining of xenograft tumors confirmed that *METTL3* KD cell‐derived tumors exhibited significantly reduced tumor size and increased TMZ sensitivity (Figure 3H). There was no significant change in the body weights of the experimental animals in each group (Figure S3G). Overall, these results support the oncogenic role of *METTL3* in GBM development and TMZ resistance.

### 3.4 METTL3 modulates m^6^A methylation of histone modification‐related genes

**FIGURE 4:**
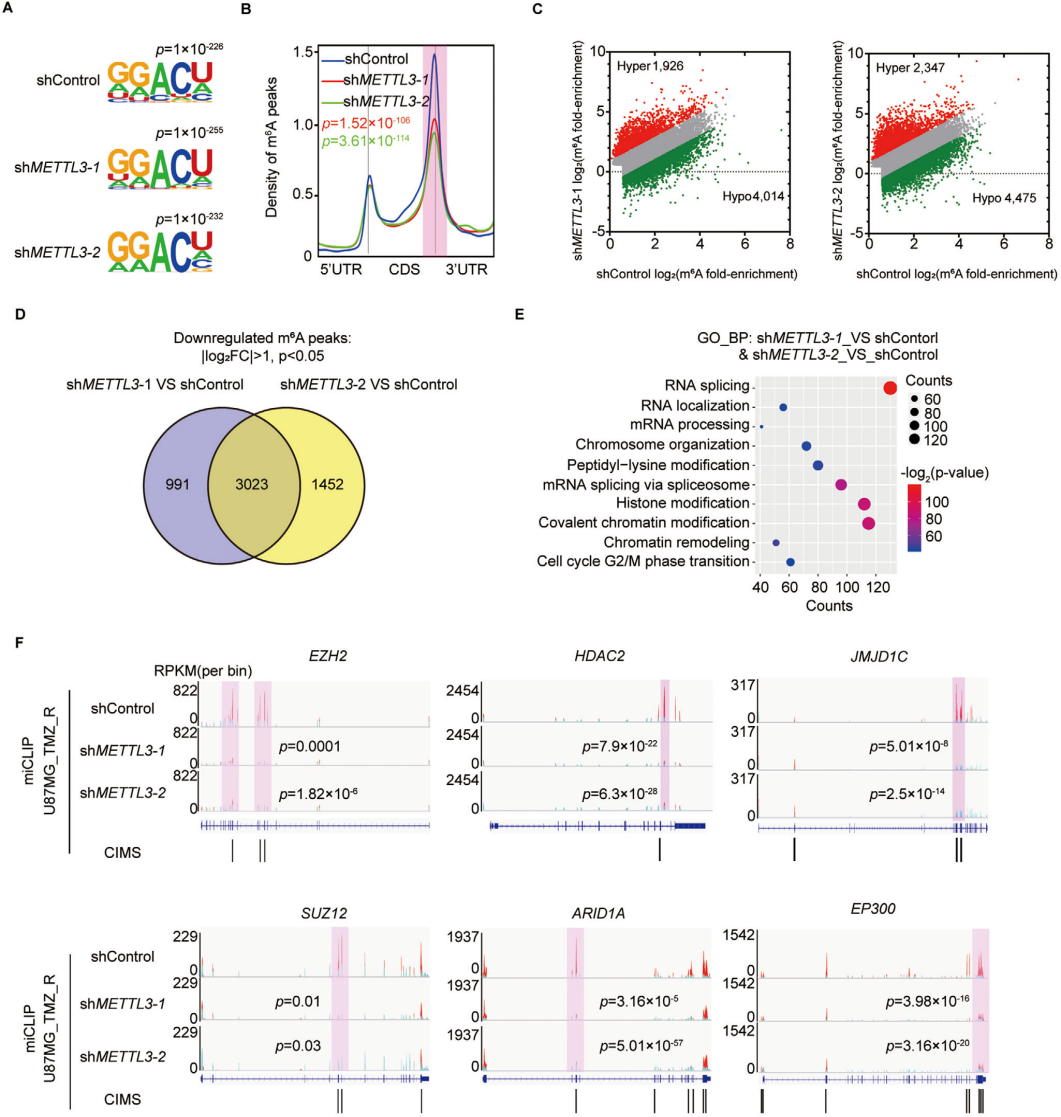
METTL3 regulates the m^6^A level of histone modification factors. (A) Motif analysis of m^6^A modification peaks in *METTL3* KD and control U87MG_TMZ_R cell miCLIP‐seq data. (B) Distribution of m^6^A modification peak reads across all mRNAs in *METTL3* KD and control U87MG_TMZ_R cells. The levels of m^6^A modification near the stop codon (shadow) in METTL3 KD and control U87MG_TMZ_R cell were compared by Student's *t*‐test. (C) Scatter plot shows m^6^A enrichment on mRNAs in *METTL3* KD and control U87MG_TMZ_R cells. (D) Venn diagram indicates the shared 3023 genes with decreased m^6^A modification in sh*METTL3*‐treated U87MG_TMZ_R cell. (E) GO analysis of m^6^A modification reduced genes in U87MG_TMZ_R cells upon *METTL3* silencing. (F) IGV plots of m^6^A peaks at the gene loci of histone modifiers in *METTL3* KD and control U87MG_TMZ_R cells. The *y*‐axis shows the normalized RPKM (per bin, bin = 25 bp) value. Exomepeak R package was used for statistical comparison. CIMS, crosslinking‐induced mutation sites

To explore the underlying mechanism of METTL3 in the regulation of TMZ resistance in GBM, we performed miCLIP‐seq on U87MG_TMZ_R cells with or without *METTL3* silencing. We identified RRACH motifs on mRNAs with m^6^A modifications across the transcriptome in each sample (Figure 4A). Consistent with previous reports,^9^ m^6^A modifications were abundant around the start and stop codons, and the majority of m^6^A peaks were distributed in the 3′ UTR and CDS regions (Figures 4B and S4A). *METTL3* KD significantly reduced the levels of m^6^A modification near the stop codon (Figure 4B). There were 3023 m^6^A peaks containing 9780 CIMS sites (putative m^6^A residues in the transcriptome) downregulated upon *METTL3* KD (Figure 4C and D). Genes with reduced levels of m^6^A modification were found to be involved in many histone modification‐related cellular processes, such as covalent chromatin modification, histone modification, and chromosome organization (Figure 4E).

Previous studies have shown that EZH2 inhibition or *HDAC2* silencing can increase the sensitivity of GBM cells to TMZ.^7^, ^33^ miCLIP‐seq revealed highly enriched and specific m^6^A peaks on *EZH2* and *HDAC2* mRNAs, which were substantially decreased in *METTL3* KD samples (Figure 4F). Furthermore, we identified a similar pattern of m^6^A modification alterations on other histone modifier mRNAs following *METTL3* KD, such as *SUZ12*, *ARID1A*, *JMJD1C*, *SETD2*, and *ARID4B* (Figures 4F and S4B).

### 3.5 *METTL3* KD impairs mRNA expression of histone modification‐related genes

**FIGURE 5:**
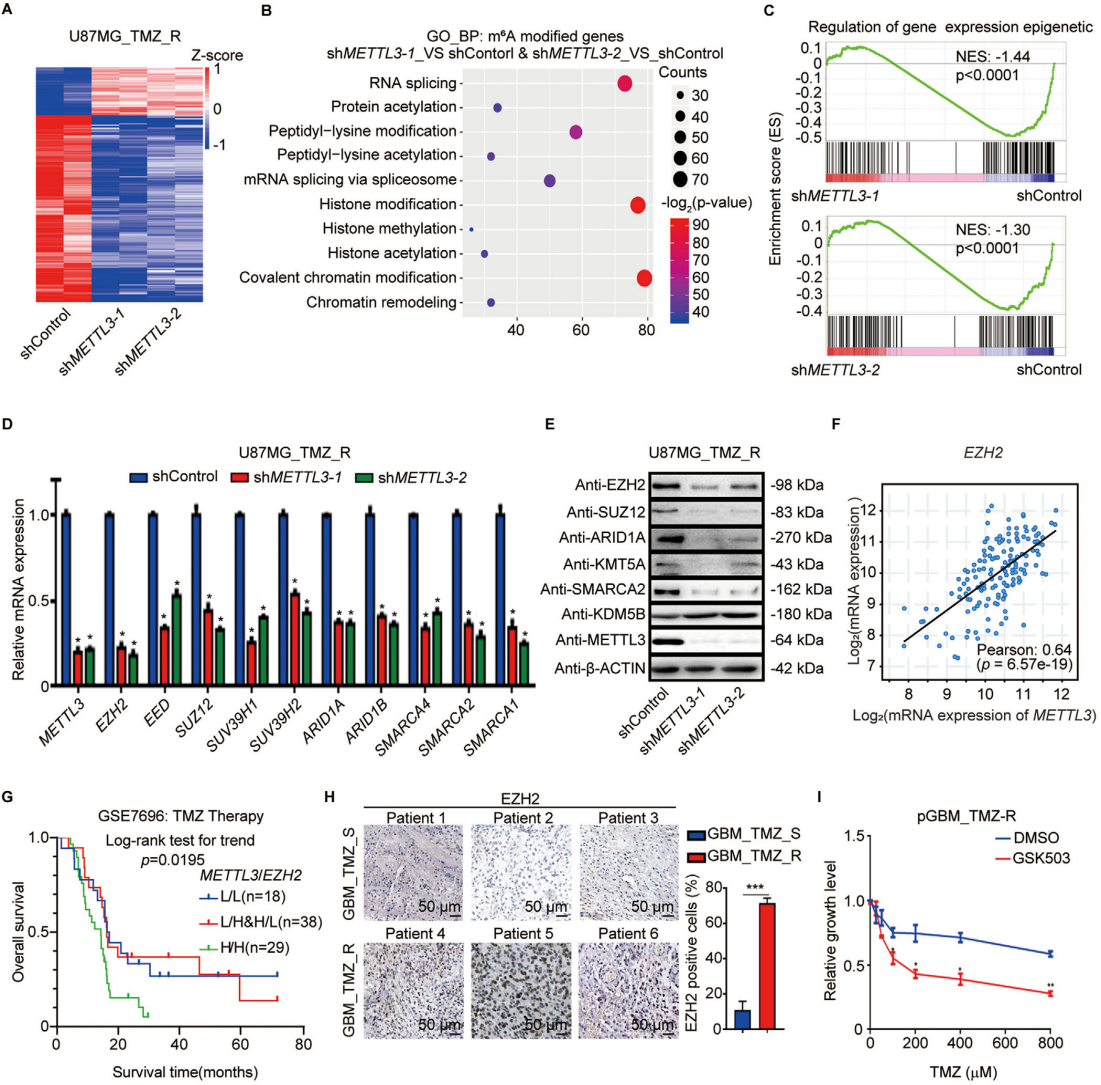
METTL3 regulates the expression of histone modification factors. (A) Heatmap shows the mRNA expression changes in U87MG_TMZ_R cells upon *METTL3* silencing. *Z*‐score = log_2_(*x*/*μ*), *μ* means the average RPKM value of a set of data. (B) GO analysis of downregulated m^6^A modified genes in U87MG_TMZ_R cells upon *METTL3* silencing. (C) GSEA plots of differentially regulated genes between sh*METTL3*‐treated and control cells. (D) RT‐qPCR analysis of the indicated mRNAs in U87MG_TMZ_R cells with or without *METTL3* silencing. (E) Immunoblotting of the indicated proteins in U87MG_TMZ_R cells transduced with sh*METTL3* and control shRNA. (F) Analysis of the correlation between *METTL3* and *EZH2* mRNA expression levels in GBM patients from TCGA database. (G) Overall survival curve of GBM patients divided by different combinations of *METTL3* and *EZH2* expression (L/L, low *EZH2* and *METTL3* expression; L/H&H/L, low *EZH2* expression and high *METTL3* expression & high *EZH2* expression and low *METTL3* expression; H/H, high *EZH2* and *METTL3* expression). (H) IHC staining of EZH2 in TMZ‐resistant GBM samples (*n* = 3) and comparison with TMZ‐sensitive GBM samples (*n* = 3). The statistical results showed the proportion of EZH2‐positive cells in each group. (I) Cell viability assays of primary TMZ‐resistant GBM cells treated with the inhibitor of *EHZ2* (GSK503) and different concentrations of TMZ. *P < 0.05; **P < 0.01, compared to control (Student's *t*‐test). All the results were obtained from three independent experiments. Values are presented as mean ± SD

To comprehensively understand the regulatory role of m^6^A modification in the expression of histone modifiers, we characterized the expression profiles of transcripts with m^6^A sites in *METTL3* KD U87MG_TMZ_R cells by RNA‐sEquation (Figure 5A). GO analysis indicated that the downregulated m^6^A modified genes were mainly related to histone modification (Figure 5B). GSEA results also revealed that the expression of genes related to epigenetic regulation in *METTL3* KD samples was significantly lower than that in the control group (Figure 5C). Indeed, there was a significant dysregulation of histone modification in *METTL3* KD TMZ‐resistant cells (Figure S5A).

We then selected several histone modification‐related genes, such as *EZH2*, *SUZ12*, and *ARID1A*, for validation because these genes play important roles in epigenetic reprogramming and/or are well‐established in sustaining drug resistance and tumorigenesis. We initially utilized real‐time PCR and immunoblotting to confirm that *METTL3* KD can remarkably decrease the expression of histone modifiers in both TMZ‐resistant (Figure 5D and E) and ‐sensitive GBM cells (Figure S5B and S5C). Consistently, histone modifiers, such as EZH2, were significantly positively correlated with METTL3 expression across the GBM patient cohort and GBM cells (Figures 5F, S5D, and S5E). As shown in Figures 5G and S5F, high expression of EZH2 and METTL3 shortened the overall survival of GBM patients.

To verify the role of EZH2 in mediating TMZ resistance in GBM, we examined EZH2 expression in human GBM samples with or without TMZ resistance. The expression of EZH2 in TMZ‐resistant samples was significantly higher than that in TMZ‐sensitive samples (Figure 5H). To enable our findings to be translated into clinical treatment, we used a novel EZH2 inhibitor, GSK503, to treat TMZ‐resistant GBM tumors. As expected, GSK503‐treated GBM cells were more sensitive to TMZ (Figure 5I). Therefore, our study identified METTL3 and EZH2 as therapeutic targets for the treatment of TMZ‐resistant GBM.

### 3.6 METTL3 regulates *EZH2* mRNA NMD in an m^6^A‐dependent manner

**FIGURE 6:**
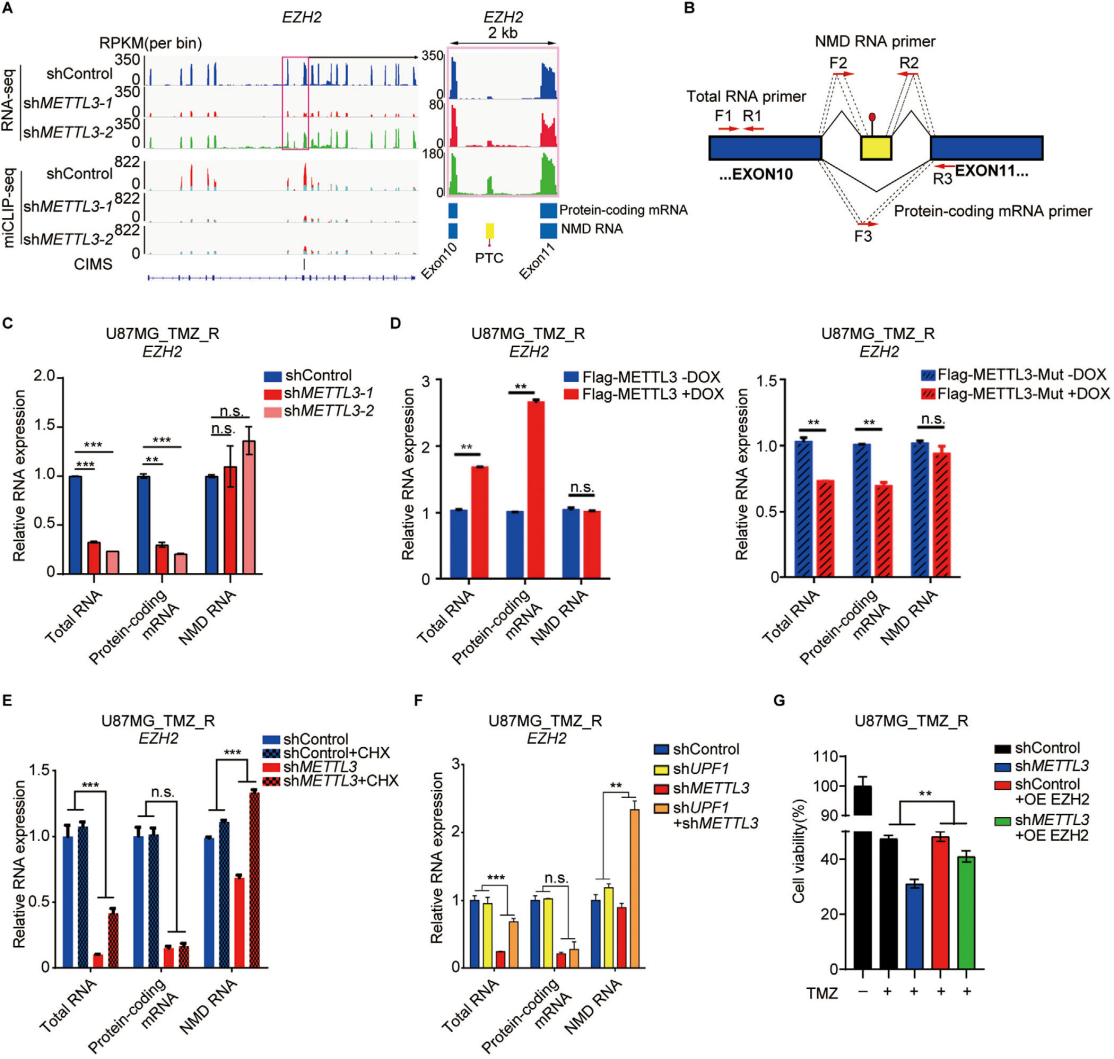
METTL3‐mediated NMD regulates EZH2 expression. (A) IGV plots of m^6^A and RNA‐seq peaks at *EZH2* mRNAs. The *y*‐axis shows the normalized RPKM (per bin, bin = 25 bp) value, blue boxes represent protein‐coding exons, and yellow boxes represent NMD exons. (B) Schematic diagrams of total RNA primers (F1 and R1), protein‐coding mRNA primers (F3 and R3), and NMD RNA primers (F2 and R2) for real‐time qPCR analyses. (C) RT‐qPCR analysis of the total, protein‐coding, or NMD RNA levels of *EZH2* in U87MG_TMZ_R cells transduced with sh*METTL3*. (D) RT‐qPCR analysis of the total, protein‐coding, or NMD RNA levels of *EZH2* in U87MG_TMZ_R cells transduced with METTL3 or a mutated catalytic domain (METTL3‐mut). (E) RT‐qPCR analysis of the total, protein coding, or NMD RNA levels of *EZH2* in *METTL3* KD (pooled *METTL3* shRNAs) U87MG_TMZ_R cells treated with 10 μg/mL CHX (cycloheximide) or DMSO for 8 h (two‐way ANOVA). (F) RT‐qPCR analysis of the total, protein coding, or NMD RNA levels of *EZH2* in U87MG_TMZ_R cells transduced with indicated shRNA(s) (pooled shRNAs) (two‐way ANOVA). (G) Cell viability assays of TMZ‐resistant U87MG cells cotransfected with indicated shRNA (pooled shRNAs) and/or overexpression plasmid and treated with TMZ (two‐way ANOVA). *P < 0.05; **P < 0.01; ***P < 0.001; And n.s., no significant difference, compared to control (Student's *t*‐test and two‐way ANOVA). All the results were obtained from three independent experiments. Values are presented as mean ± SD

Next, we sought to elucidate the mechanism by which m^6^A regulates the expression of histone modification‐related genes. We combined RNA‐seq and miCLIP‐seq data of the *EZH2* locus, and found that the ratio of the NMD isoform of *EZH2* transcript increased when the m^6^A modification decreased upon *METTL3* KD (Figure 6A). We also observed an increase in the expression of other histone modification‐related genes with m^6^A modification in TMZ‐resistant GBM (Figure S6A). We designed primers that specifically targeted *EZH2* NMD RNA, *EZH2* protein‐coding mRNA, and *EZH2* total RNA (Figure 6B). RT‐qPCR results showed that the levels of protein‐coding mRNA of *EZH2* decreased whereas that of the NMD RNA remained almost unchanged because the RNA in the form of NMD was degraded continuously and could not accumulate in the cells (Figure 6C). Conversely, overexpression of wild‐type METTL3, but not mutant METTL3 (METTL3‐Mut; a catalytically inactive mutant), significantly enhanced the levels of total and protein‐coding mRNA of *EZH2* (Figure 6D).

To observe the accumulation of *EZH2* NMD RNA after *METTL3* KD, we used cycloheximide or silenced *UPF1* (a core factor of NMD) to inhibit the NMD pathway. Once NMD was inhibited, increased *EZH2* NMD RNA levels were observed in *METTL3* KD U87MG_TMZ_R cells (Figure 6E and F). Moreover, the inhibitory effects of *METTL3* KD on GBM cell growth were partially rescued by the forced expression of EZH2 (Figure 6G). Taken together, these findings indicate that m^6^A promotes EZH2 expression by suppressing NMD.

### 3.7 EZH2 is an important regulator of METTL3 in GBM

**FIGURE 7:**
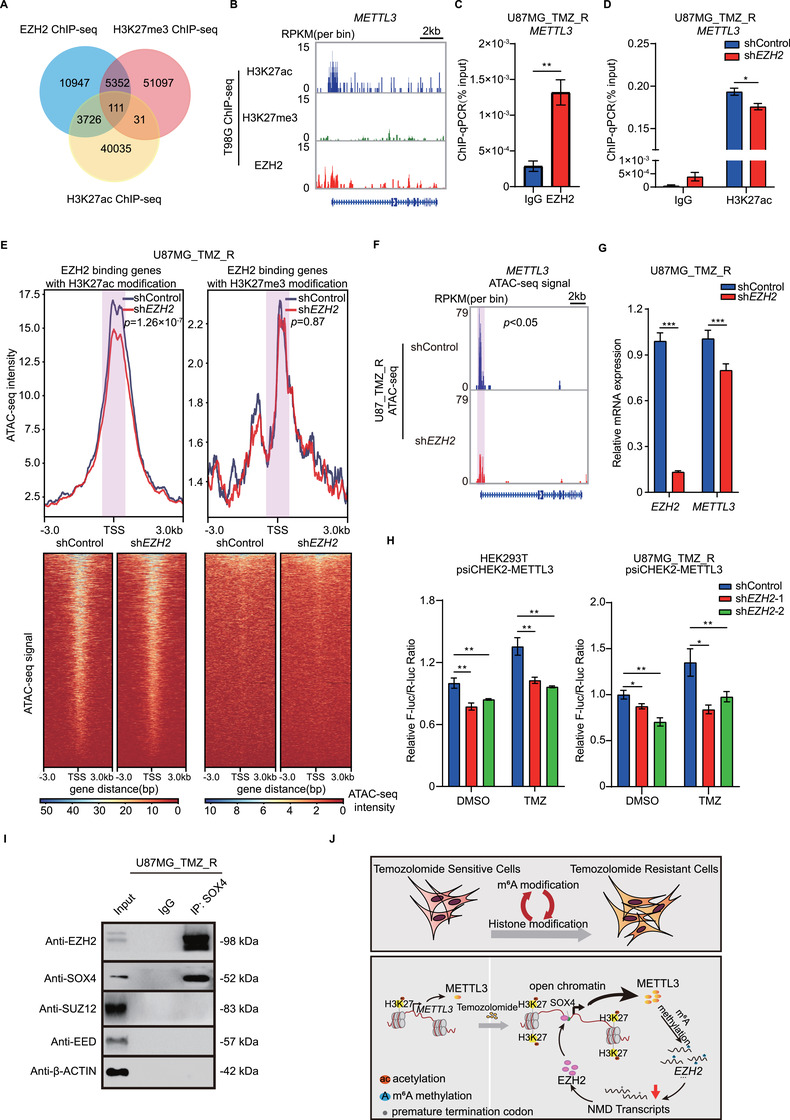
EZH2‐mediated H3K27ac enhances *METTL3* locus accessibility in GBM cells. (A) Venn diagram illustrates overlap among EZH2, H3K27ac, and H3K27me3 binding sites. ChIP‐seq data were acquired from GSE128275 and GSE112240. (B) *METTL3* promoter is occupied by EZH2 and H3K27ac but not by H3K27me3. ChIP‐seq data were acquired from GSE128275 and GSE112240. The *y*‐axis shows the normalized RPKM (per bin, bin = 25 bp) value. (C) ChIP‐qPCR analysis of EZH2 enrichment at the *METTL3* promoter region in U87MG_TMZ_R cells. (D) ChIP‐qPCR analysis of H3K27ac enrichment at the *METTL3* promoter region in *EZH2* KD (pooled *EZH2* shRNAs) or control U87MG_TMZ_R cells. (E) Average intensities of ATAC‐seq signals in *EZH2* KD (pooled *EZH2* shRNAs) or control U87MG_TMZ_R cells. The ATAC‐seq data of EZH2‐binding genes with H3K27ac modification and EZH2‐binding genes with H3K27me3 modification were analyzed. ATAC‐seq signal around TSSs (shadow) were compared by Student's *t*‐test. (F) IGV plots of ATAC‐seq peaks at *METTL3* mRNAs in U87_TMZ_R cells with or without *EZH2* KD (pooled *EZH2* shRNAs). The *y*‐axis shows the normalized RPKM (per bin, bin = 25 bp) value. ATAC‐seq signal around TSS of METTL3 (shadow) was compared by MACS2. (G) mRNA expression of *EZH2* and *METTL3* in *EZH2* KD (pooled *EZH2* shRNAs) or control U87MG_TMZ_R cells. (H) Dual‐luciferase reporter assay for the effects of *EZH2* KD on the luciferase activity of the *METTL3* promoter (–3000 bp‐0 bp) in HEK293T and U87MG_TMZ_R cells. (I) Co‐IP analysis of the interaction between SOX4 and PRC2 complex components in U87MG_TMZ_R cells. (J) Schematic illustration of the working model. *P < 0.05; **P < 0.01; and ***P < 0.001, compared to control (Student's *t*‐test). All the results were obtained from three independent experiments. Values are presented as mean ± SD. n.s., no significant difference

EZH2 is known to act as a transcription repressor, which can establish trimethylation at lysine 27 of histone H3 (H3K27me3), a repressive mark for gene expression.^34^ However, a recent study revealed that EZH2 could play a dual role in regulating gene expression.^34^, ^35^, ^36^ To further characterize the oncogenic function of EZH2 in GBM, we analyzed EZH2 ChIP‐seq and H3K27me3/H3K27ac ChIP‐seq data from published datasets (GSE128275 and GSE112240). EZH2 ChIP‐seq revealed that 5463 gene loci were significantly enriched with H3K27me3, while 3837 gene loci were enriched with H3K27ac. Only 111 gene loci overlapped in the H3K27me3 ChIP‐seq and H3K27ac ChIP‐seq data (Figure 7A). Intriguingly, the *METTL3* locus was bound by EZH2 and enriched with H3K27ac but not H3K27me3 (Figure 7B), suggesting that EZH2 may act as an activator of *METTL3* expression in an H3K27ac‐dependent manner. We confirmed via the ChIP‐qPCR assay that EHZ2 bound to the *METTL3* locus in U87MG_TMZ_R cells (Figure 7C). KD of *EZH2* reduced H3K27ac modification on METTL3 promoter in TMZ‐resistant U87MG cells (Figure 7D).

Considering that open chromatin regions were enriched with H3K27ac, we investigated whether *EZH2* KD could affect the chromatin accessibility of gene loci with H3K27ac. An ATAC‐seq assay was performed (Figure S7A) and it was observed that *EZH2* KD significantly reduced the signal of ATAC‐seq peaks in regions with H3K27ac, but not with H3K27me3 (Figure 7E). GO analysis showed that these H3K27ac‐dependent genes were mainly enriched in the metabolic process signaling pathways (Figure S7B). Consistently, chromatin accessibility of the *METTL3* locus were significantly decreased upon *EZH2* KD in U87MG_TMZ_R cells (Figure 7F). *EZH2* KD in GBM cells reduced *METTL3* mRNA expression (Figure 7G) and luciferase reporter activity upon TMZ treatment (Figure 7H). Furthermore, *METTL3* expression was significantly higher in GBM with high expression of both *SOX4* and *EZH2* than in GBM with low expression of *SOX4* and/or *EZH2* (Figure S7C).

Next, we observed that SOX4 interacted with EZH2, but not with SUZ12 or EED, suggest that SOX4 and EZH2 may form a co‐activator complex at the *METTL3* locus (Figure 7I). Moreover, ChIP‐qPCR results revealed that the binding of SOX4 to the *METTL3* promoter was significantly reduced when *EZH2* was knocked down (Figure S7D). In turn, *SOX4* KD also affected the binding of EZH2 to the *METTL3* promoter (Figure S7D). Taken together, these findings suggest that EZH2 and SOX2 directly bound to *METTL3* promoter to facilitate the transcription activation in an H3K27me3 independent manner.

## 4 DISCUSSION

Although TMZ has been demonstrated as the first‐line chemotherapy agent for patients with GBM, acquired resistance is a major obstacle to its clinical efficacy. Hence, there is a high demand for new targets that play key roles in the regulation of TMZ resistance. In this study, we demonstrated that m^6^A modulates the NMD of histone modifiers in GBM, thus endowing chromatin remodeling and TMZ resistance in GBM cells (Figure 7J, upper). We also revealed that, after TMZ treatment, the level of METTL3‐mediated m^6^A modification increased owing to the upregulation of EZH2/SOX4 expression and enhanced chromatin accessibility (Figure 7J, lower). Overall, understanding how m^6^A confers TMZ resistance in GBM cells may help develop novel therapeutic interventions.

Our study uncovers the crosstalk between m^6^A RNA modification and histone modification in the context of TMZ resistance. This crosstalk has been found in many biological processes, however, the mechanism remains largely unclear. It has been reported that *METTL14* knockout leads to a genome‐wide increase in histone modification, including H3K27me3, H3K27ac, and H3K4me3.^37^ Our previous study uncovered a direct role of KDM6B in promoting METTL3/METT14 recruitment and m^6^A deposition during inflammatory responses.^38^ At present, our findings suggested that TMZ exposure leads to changes in chromatin accessibility at the *METTL3* locus, thereby increasing m^6^A modification in GBM cells. SOX4 functions as a TF for *METTL3* and plays a key role in the regulation of promoter remodeling and Pol II recruitment in GBM cells. Importantly, TMZ treatment led to increase of m^6^A RNA modification on histone modifiers. Altered expression of histone modulators, accompanied with chromatin structure remodeling, can lead to transcriptional plasticity in tumor cells, thereby driving their transformation toward an adaption state.^39^ These “reprogrammed” cells can either reexpand when TMZ treatment is discontinued or acquire permanent resistance of TMZ.

Targeting m^6^A has shown great potential in overcoming drug resistance in different cancer types.^26^, ^27^, ^40^ Although the roles of m^6^A regulator (eg, hnRNPA2 and FTO) in TMZ resistance and radioresistance have been reported, there are many major knowledge gaps that remain to be filled. Whether the m^6^A methylation position and the level of m^6^A methylation in RNA transcripts modulate TMZ resistance in GBM remains elusive. To address this question, we, for the first time, performed miCLIP‐seq to map m^6^A locations with single‐nucleotide resolution in TMZ‐resistant and ‐sensitive clinical GBM samples. These data are helpful in identifying key m^6^A methylation sites that regulate TMZ resistance. In addition, the m^6^A modification participates in various complex biological processes, indicating that m^6^A may also play a role in chemotherapeutics other than TMZ. Thus, a comprehensive understanding of the m^6^A functions in chemoresistance should facilitate the development of new therapeutic strategies to overcome drug resistance and enhance therapeutic efficacy.

Our study also elucidated the regulatory molecular mechanism of m^6^A in stabilizing the mRNAs of histone modification‐related genes by preventing NMD, as a proof of concept, the prevention of NMD of the histone methyltransferase EZH2. EZH2 is an enzymatic catalytic subunit of PRC2 that can suppress downstream gene expression by depositing H3K27me3 modifications. EZH2 can also regulate gene expression in an H3K27me3‐independent manner.^34^, ^35^, ^36^ In our study, EZH2 bound to the *METTL3* promoter and marked it with H3K27ac, leading to gene activation. EZH2 contributes to TMZ resistance in gliomas and is essential for GSC phenotypes. Han et al reported that EZH2 epigenetically regulated the FADD/PARP1 axis, leading to TMZ resistance in glioma.^7^ Additionally, EZH2 inhibitors reduced primary GBM cell viability when combined with TMZ and impaired GSC self‐renewal and tumor‐initiating capacity.^41^

Intriguingly, our study revealed that METTL3‐EZH2 formed a positive feedback regulatory loop, and EZH2 could promote *METTL3* expression by recruiting SOX4. Overexpression of SOX4 has been reported in GBM, however, the underlying mechanism of its actions remains elusive. Zhang et al found that SOX4 acts as a tumor suppressor in GBM by induce cell cycle arrest and cell growth inhibition.^42^ We found that SOX4 functions as a transcriptional activator for METTL3 in GBM cells. These findings allowed us to propose a strategy for the use of clinically available EZH2 inhibitors and SOX4 inhibitors to treat TMZ‐resistant GBMs with aberrant high level of m^6^A modification.

## 5 CONCLUSION

In conclusion, our study revealed that m^6^A regulates histone modification by inhibiting NMD as a previously unknown mechanism of TMZ resistance in GBM patients. Our study also identified the critical targets of m^6^A, suggesting that an inhibitor targeting the SOX4/EZH2/METTL3 axis may provide therapeutic benefits for GBM patients with TMZ resistance.

## CONFLICT OF INTEREST

No potential conflict of interest to disclose.

## AUTHOR CONTRIBUTIONS

F. L., S. C., and J. Y. performed the experiments and conducted bioinformatics analysis of the sequencing data. Z. G. performed ATAC‐seq analysis. Z. S., Y. Y., and T. L. prepared plasmids and lentiviral vectors. Y. P. and C. Q. interpreted the clinical data and revised the manuscript. W. L., Q. L., and W. Z. designed the experiments, interpreted the data, wrote the manuscript, and supervised the study.

## ETHICS STATEMENT

All animal experiments were approved by the Institutional Animal Care and Use Committee of Sun Yat‐Sen University (Approval No. SYSU‐IACUC‐2019‐B073) and carefully conducted according to the Guide for the Care and Use of Laboratory Animals.

## Supporting information

Supporting Information

## Data Availability

The datasets in this study are available from the corresponding author on request.
