# Supplementary material for: Interplay of m6A and histone modifications contributes to temozolomide resistance in glioblastoma
Source: Clin Transl Med. 2021 Sep 15;11(9):e553. doi: 10.1002/ctm2.553 (PMC8441140; doi:10.1002/ctm2.553)
Supplement: Supplementary file 1 — Supporting Information [file CTM2-11-e553-s001.docx]

**Supplementary Information**

**Interplay of m^6^A and histone modifications contributes to temozolomide resistance in glioblastoma**

Fuxi Li, Siyun Chen, Jiaming Yu, Zhuoxing Gao, Zhangyi Sun, Yang Yi, Teng Long, Chuanxia Zhang, Yuzhe Li, Yimin Pan, Chaoying Qin, Wenyong Long, Qing Liu, Wei Zhao

**Contents**

**Supplementary Figure 1-7**

**Supplementary Table 1-2**

**Supplementary Methods**

**Supplemental Figures**

**Supplemental Figure 1**

**
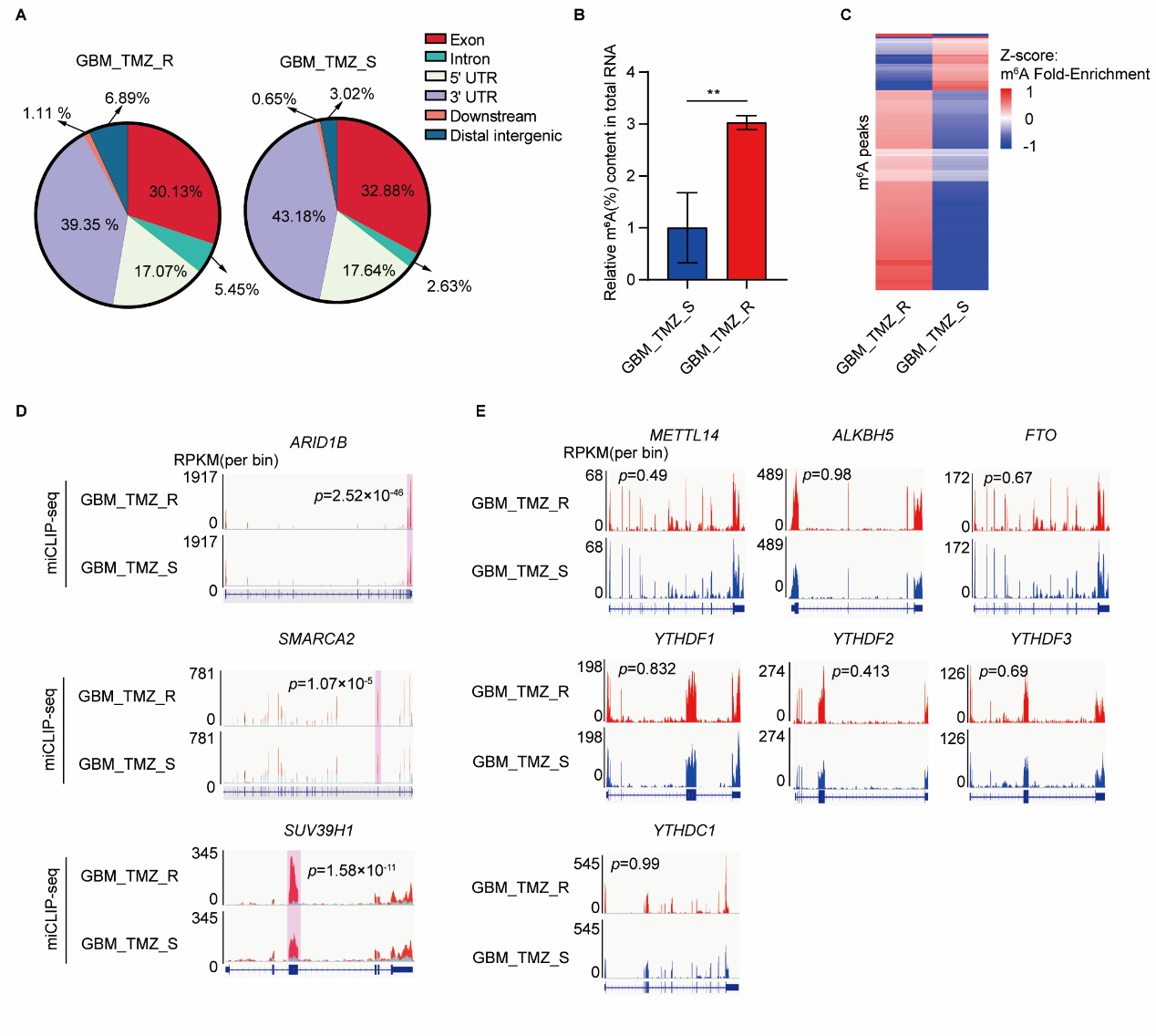
**

**Supplemental Figure 1. The difference of m^6^A methylome between TMZ resistant and sensitive GBM patient samples shows the increased expression of METTL3 in TMZ resistant GBM. Related to Figure 1.**

(A) Distribution of m^6^A modification peaks in TMZ resistant GBM tumors and TMZ sensitive GBM tumors.

(B) m^6^A levels in TMZ-resistant GBM (n=3) relative to TMZ-sensitive GBM (n=3).

(C) Heatmap showing the differential genes of m^6^A modification between TMZ resistant and sensitive GBM samples. Z-score = log_2_(x/μ), μ means the average m^6^A Fold-Enrichment value of a set of data.

(D) The m^6^A modification status of histone modification related genes *ARID1B*, *SMARCA2*, *SUV39H1* in TMZ resistant and sensitive GBM samples. The y-axis shows the nomalized RPKM (per bin, bin = 25bp) value. Exomepeak R package were used for statistical comparison. DESeq2 was used for statistical comparison.

(E) Integrative genomics viewer (IGV) plots of RNA-seq peaks at m^6^A modulators mRNAs.

**, p < 0.01, compared to control (Student’s t-test)

**Supplemental Figure 2**


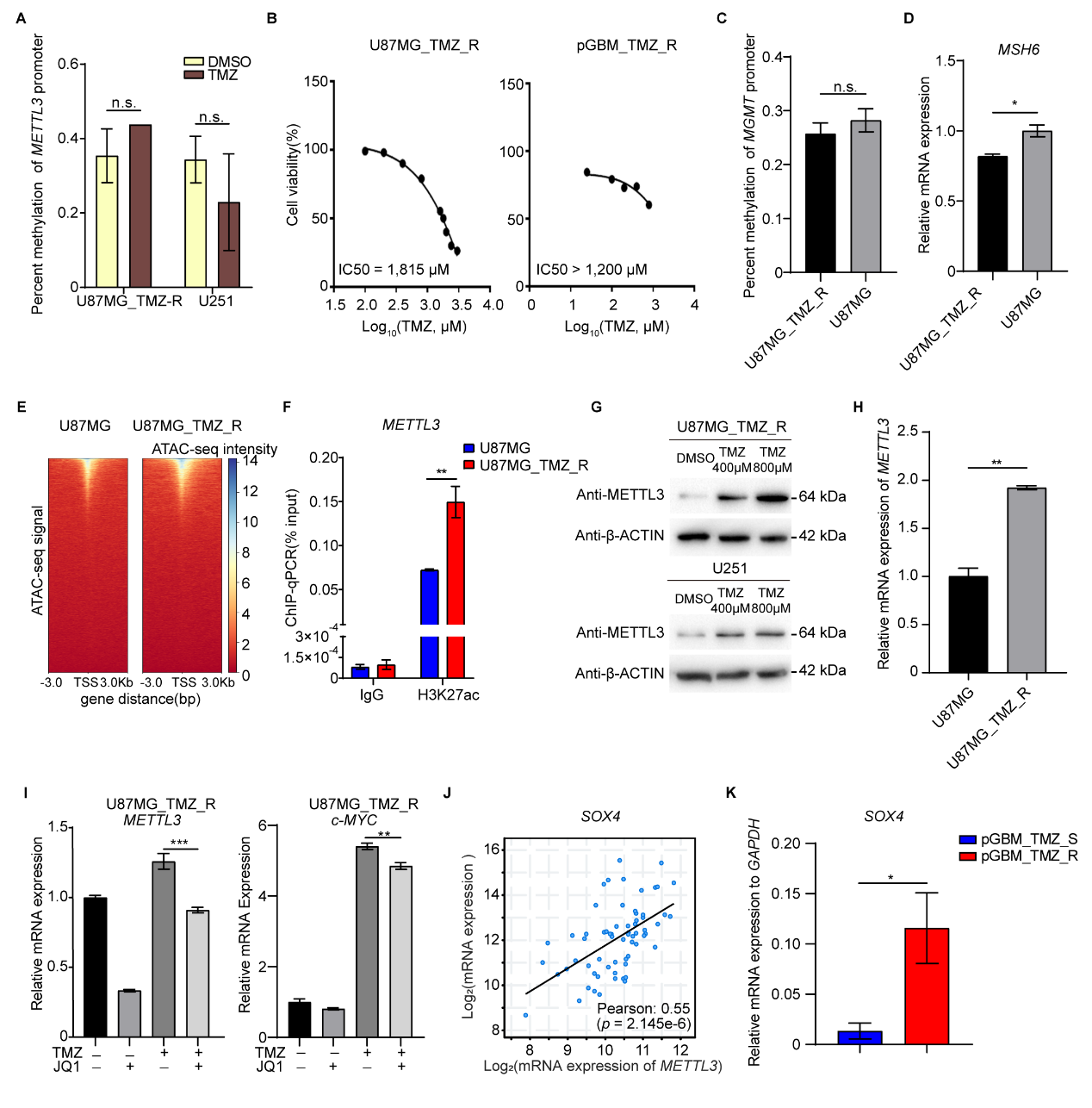


**Supplemental Figure 2. SOX4 participated in TMZ induced enhancement of transcriptional activity of *METTL3*. Related to Figure 2.**

(A) Percent methylation of *METTL3* promoter in U87MG_TMZ_R or U251 cells after treated with DMSO or TMZ.

(B) Survival curves of TMZ resistant U87MG cells (U87MG_TMZ_R) and primary TMZ resistant GBM cells (pGBM_TMZ_R) upon TMZ treatment.

(C) Percent methylation of *MGMT* promoter in U87MG_TMZ_R and U87MG cells.

(D) qRT-PCR analysis of *MSH6* expression in U87MG_TMZ_R and U87MG cells.

(E) Heatmap showing the ATAC-seq signal at TSS ± 3 kb regions for all genes in U87MG and TMZ resistant U87MG cells (U87MG_TMZ_R).

(F) ChIP-qPCR analysis of H3K27ac enrichment at the *METTL3* promoter region in U87MG and U87MG_TMZ_R cells.

(G)Western blotting indicates the expression of METTL3 in U87MG_TMZ_R and U251 cells treated with DMSO and TMZ. Cells were treated with DMSO or 400/800 μM TMZ for 72 hours. Then the cell lysates were harvested and used for detecting the expression of METTL3 by immunoblotting.

(H) Expression levels of *METTL3* were analyzed by quantitative real-time PCR (qPCR) in U87MG and U87MG_TMZ_R cells.

(I) Real-time qPCR analyses show the mRNA expression of *METTL3* and *c-MYC* in U87MG_TMZ_R cells after treating with TMZ or/ and JQ1 for 72h.

(J) Analysis of the correlation between *METTL3* and *SOX4* mRNA expression levels in GBM patients from TCGA database.

(K) Expression levels of *SOX4* were analyzed by quantitative real-time PCR (qPCR) in TMZ-resistant (n=3) and -sensitive (n=3) GBM samples.

*, *p* < 0.05, **, *p* < 0.01, ***, *p* < 0.001 are based on the Student’s t-test. Values are mean ± SD of three independent experiments.

**Supplemental Figure 3**


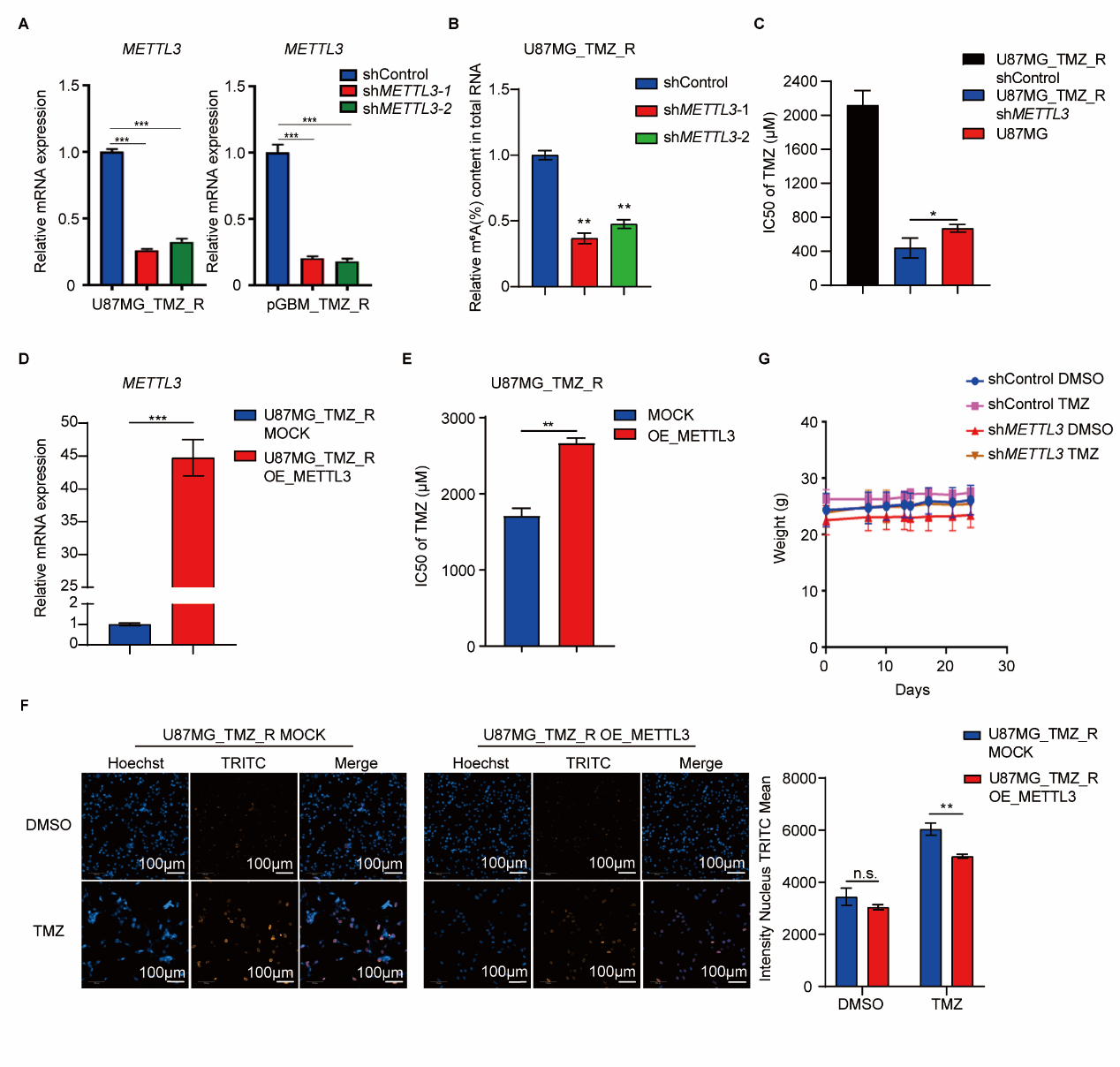


**Supplemental Figure 3. METTL3 inhibition enhances sensitivity of TMZ resistant GBM cells to TMZ. Related to Figure 3**.

(A) Real-time qPCR analyses indicate a significant down-regulation of *METTL3* in METTL3-KD U87MG_TMZ_R and pGBM_TMZ_R.

(B) m^6^A levels in shMETTL3-1 and shMETTL3-2 TMZ resistant U87MG cells (U87MG_TMZ_R) relative to control shRNA.

(C) The IC50 of TMZ in U87MG_TMZ_R and pGBM_TMZ_R cells transduced with shMETTL3 and control shRNA.

(D) qRT-PCR demonstrated a significant up-regulation of *METTL3* in OE-METTL3 U87MG_TMZ_R cells.

(E) TMZ resistant U87MG cells were transfected with MELLT3-overexpressing vector (OE_METTL3) or control vector (MOCK) before they were treated with the indicated concentrations of TMZ for 72 h. The IC50 of TMZ in the two groups is shown.

(F) DNA damage assay indicated overexpression of METTL3 protected TMZ resistant U87MG cells from DNA damage by TMZ. High intensity of TRITC signal means strong damage.

(G) Body weight growth curve in four group mice.

*, *p* < 0.05, **, *p* < 0.01, ***, *p* < 0.001 are based on the Student’s t-test. Values are mean ± SD of three independent experiments.

**Supplemental Figure 4**


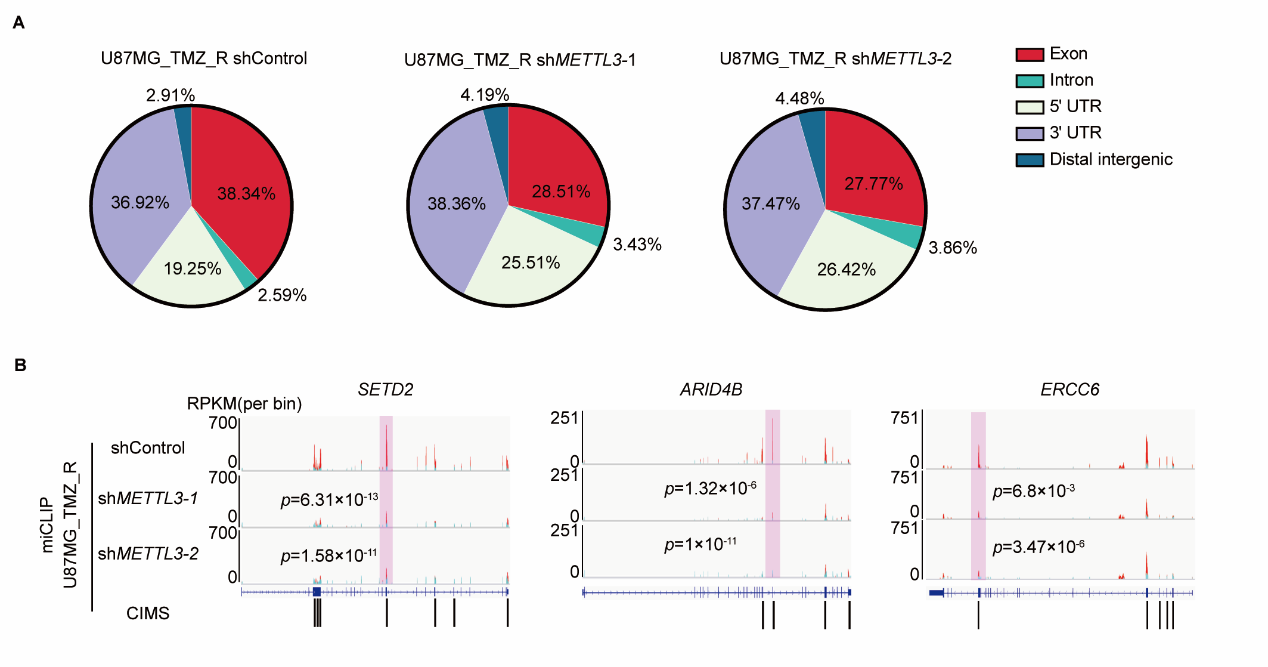


**Supplemental Figure 4. METTL3 regulates the m^6^A level of histone modification factors. Related to Figure 4.**

(A) Pie charts demonstrate the m^6^A peaks distribution in control and *METTL3* KD U87MG_TMZ_R cells.

(B) Integrative genomics viewer (IGV) plots of m^6^A peaks at histone modification factors mRNAs upon *METTL3* silencing. The y-axis shows the nomalized RPKM (per bin, bin = 25bp) value. Exomepeak R package was used for statistical comparison.

**, p < 0.01, is based on the Student’s t-test. Values are mean ± SD of three independent experiments.

**Supplemental Figure 5**


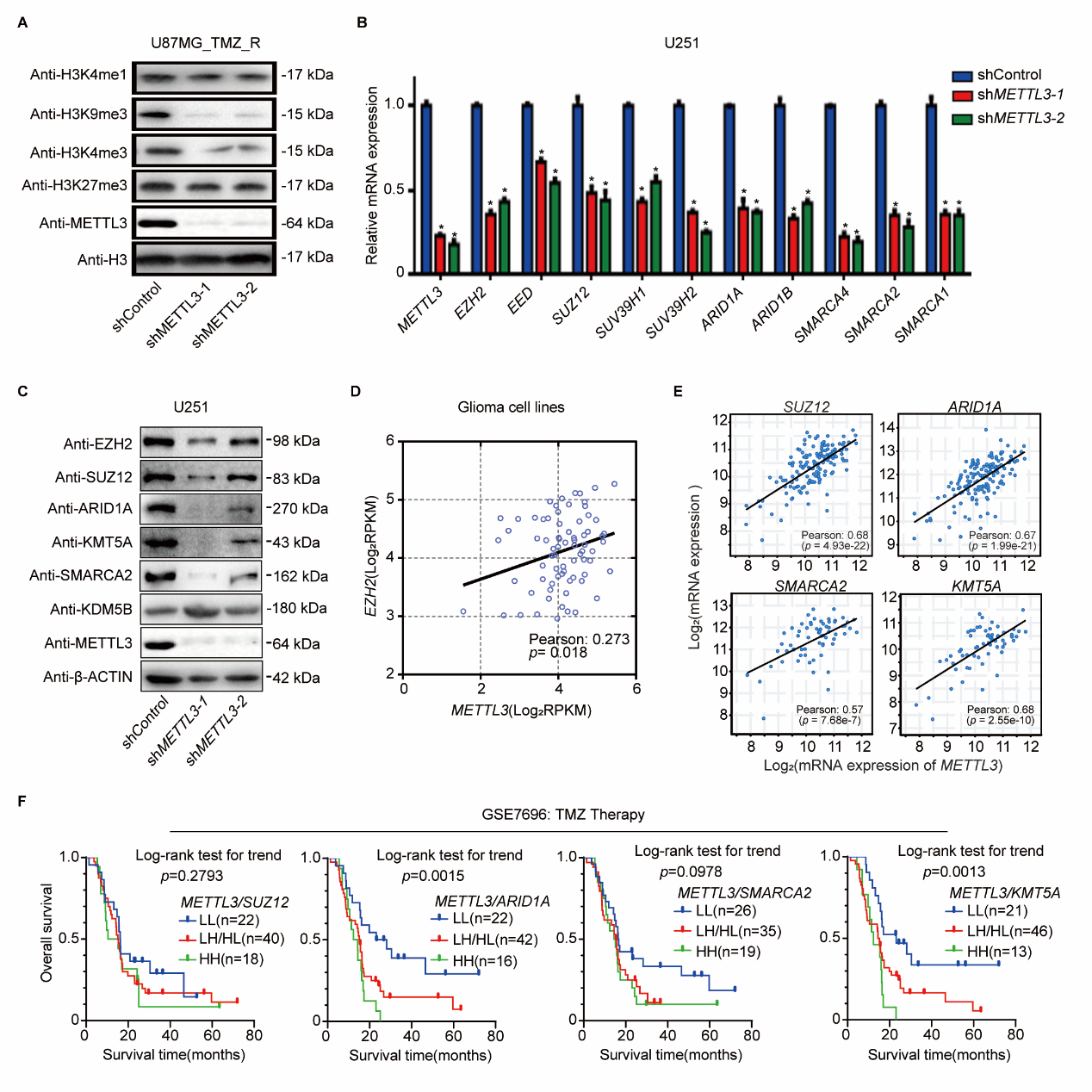


**Supplemental Figure 5. METTL3 regulates the expression of histone modification factors. Related to Figure 5.**

(A) Immunoblotting of the indicated proteins in U87MG_TMZ_R cells transduced with sh*METTL3* and control shRNA.

(B) RT-qPCR analysis of the indicated mRNAs in U251 cells with or without *METTL3* silencing.

(C) Immunoblotting of the indicated proteins in U251 transduced with sh*METTL3* and control shRNA.

(D) Analysis of the correlation between *METTL3* and *EZH2* mRNA expression levels in Glioma cell lines from TCGA database.

(E) Analysis of the correlation between *METTL3* and histone modification factors (*SUZ12*, *ARID1A*, *SMARCA2*, and *KMT5A*) mRNA expression levels in GBM patients from TCGA database.

(F) The overall survival curve of GBM patients (dataset from GSE7696) divided by combination of *METTL3* and histone modification factors (*SUZ12*, *ARID1A*, *SMARCA2*, and *KMT5A*) expression.

*, *p* < 0.05, **, *p* < 0.01, ***, *p* < 0.001 are based on the Student’s t-test. Values are mean ± SD of three independent experiments.

**Supplemental Figure 6**


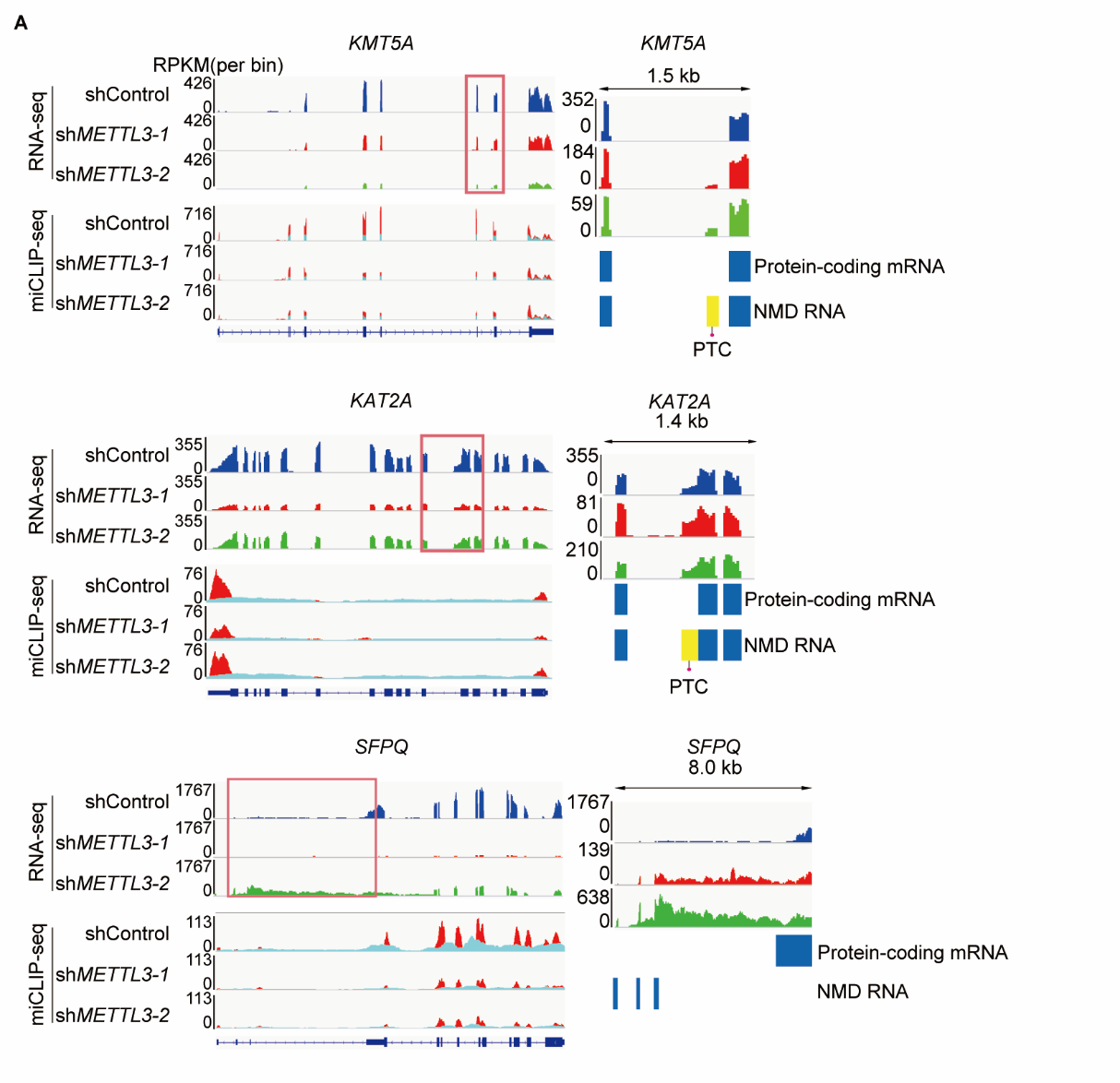


**Supplemental Figure 6.** **METTL3-mediated NMD regulates EZH2 expression. Related to Figure 6.**

(A) Integrative genomics viewer (IGV) plots of m6A peaks and RNA-seq peaks at *KMT5A*, *KAT2A*, *SFPQ* mRNAs. The y-axis shows the nomalized RPKM (per bin, bin = 25bp) value, blue boxes represent protein-coding exons, and yellow boxes represent NMD exons.

**Supplemental Figure 7**


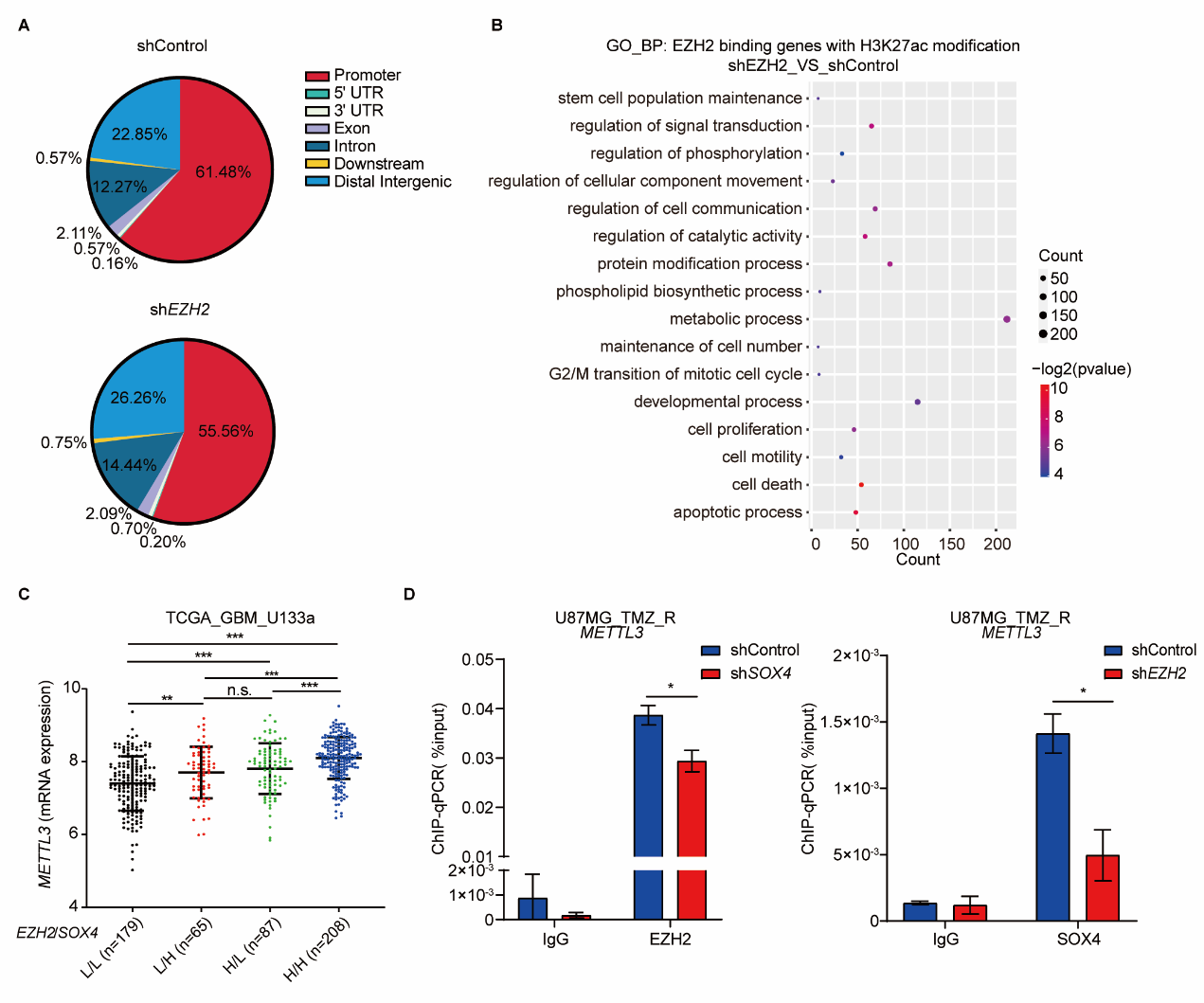


**Supplemental Figure 7.** **EZH2-mediated H3K27ac enhances *METTL3* locus accessibility in GBM cells. Related to Figure 7.**

1. Pie charts show the ATAC-seq signal distribution of EZH2 binding genes in control and *EZH2* KD U87MG_TMZ_R cells.
2. GO analysis of ATAC-seq differential EZH2 binding genes with H3K27ac modification in *EZH2*-KD or control U87MG_TMZ_R cells.
3. Comparison of METTL3 expression in the 4 subsets. (mRNA expression of *EZH2* and *SOX4* in GBM patients from TCGA_GBM_U133a). mRNA expression values are divided in 4 groups according to their distribution relative to the mean values for *EZH2* and *SOX4*: H/H (high *EZH2* and high *SOX4*; blue), L/H (low *EZH2* and high *SOX4*; red), H/L (high *EZH2* and low *SOX4*; green) and L/L (low *EZH2* and low *SOX4*; black).
4. ChIP-qPCR analysis of SOX4 enrichment at the *METTL3* promoter region in *EZH2* KD or control U87MG_TMZ_R cells was showed on the left. ChIP-qPCR analysis of EZH2 enrichment at the METTL3 promoter region in *SOX4* KD or control U87MG_TMZ_R cells was showed on the right.

*, *p* < 0.05, **, *p* < 0.01, ***, *p* < 0.001 are based on the Student t test. Values are mean ± SD of three independent experiments.

**Supplementary Tables**

**Supplementary Table 1. shRNA sequences used for experiments in this study**

| Names | Sequences |
| --- | --- |
| Control shRNA | CAACAAGATGAAGAGCACCAA |
| *METTL3* shRNA-1 | GCTGCACTTCAGACGAATTAT |
| *METTL3* shRNA-2 | GCCAAGGAACAATCCATTGTT |
| *UPF1* shRNA-1 | CCAACCCGATAAACCGATGTT |
| *UPF1* shRNA-2 | CCTGCGTGGTTTACTGTAATA |
| *SOX4* shRNA-1 | TGGGCACATCAAGCGACCCAT |
| *SOX4* shRNA-2 | AGCGACAAGATCCCTTTCATT |
| *EZH2* shRNA-1 | TTGGGACAGTAAAAATGTGTC |
| *EZH2* shRNA-2 | GTTTAGAGTCAAAGAATCTAG |

**Supplementary Table 2. Sequences of primers used for experiments in this study**

| Names | Sequences |
| --- | --- |
| human *METTL3*-forward | CATTGCCCACTGATGCTGTG |
| human *METTL3*-reverse | AGGCTTTCTACCCCATCTTGA |
| human *SOX4*-forward | CCTTAACCTGCCACCAGTGT |
| human *SOX4*-reverse | AGCCTCTGGACAGAGCAGTA |
| human *UPF1*-forward | CTGCAACGGACGTGGAAATAC |
| human *UPF1*-reverse | ACAGCCGCAGTTGTAGCAC |
| human *EZH2*-forward | GTACACGGGGATAGAGAATGTGG |
| human *EZH2*-reverse | GGTGGGCGGCTTTCTTTATCA |
| human *EED*-forward | GTGACGAGAACAGCAATCCAG |
| human *EED*-reverse | TATCAGGGCGTTCAGTGTTTG |
| human *SUZ12*-forward | AGGCTGACCACGAGCTTTTC |
| human *SUZ12*-reverse | GGTGCTATGAGATTCCGAGTTC |
| human *SUV39H1*-forward | CCTGCCCTCGGTATCTCTAAG |
| human *SUV39H1*-reverse | ATATCCACGCCATTTCACCAG |
| human *SUV39H2*-forward | TCTATGACAACAAGGGAATCACG |
| human *SUV39H2*-reverse | GAGACACATTGCCGTATCGAG |
| human *ARID1A*-forward | CCTGAAGAACTCGAACGGGAA |
| human *ARID1A*-reverse | TCCGCCATGTTGTTGGTGG |
| human *ARID1B*-forward | GGCCGTCCCGGAGTTTAATAA |
| human *ARI1DB*-reverse | CGGAGTGCATCATCCCCAT |
| Names | Sequences |
| human *SMARCA4*-forward | AATGCCAAGCAAGATGTCGAT |
| human *SMARCA4*-reverse | GTTTGAGGACACCATTGACCATA |
| human *SMARCA2*-forward | AGGGGATTGTAGAAGACATCCA |
| human *SMARCA2*-reverse | TTGGCTGTGTTGATCCATTGG |
| human *SMARCA1*-forward | GATGCGACCGCCACTATCG |
| human *SMARCA1*-reverse | ATTTAGGCGCTTTAGCAGCAA |
| *EZH2*-Total-forward | GTACACGGGGATAGAGAATGTGG |
| *EZH2*-Total-reverse | GGTGGGCGGCTTTCTTTATCA |
| *EZH2*-protein coding mRNA-forward | CAGCATTTGGAGGGAGCA |
| *EZH2*-protein coding mRNA-reverse | GCTGGGCCTGCTACTGTTATT |
| *EZH2*-NMD RNA-forward | CAGCATTTGCCACTCCTACC |
| *EZH2*-NMD RNA-reverse | AGAGCAGCAGCAAACTCCTTT |
| H3K27ac ChIP-qPCR-forward | GCGCCTTATTCGAGAGGTGT |
| H3K27ac ChIP-qPCR-reverse | CTTGTGGGCCTGGATAGAGC |
| SOX4 ChIP-qPCR-forward | GCACACCAAAATGTTCAGCT |
| SOX4 ChIP-qPCR-reverse | GTACGTGTAATCTATTAAAATCG |
| Pol Ⅱ ChIP-qPCR-forward | GCGCCTTATTCGAGAGGTGT |
| Pol Ⅱ ChIP-qPCR-reverse | CTTGTGGGCCTGGATAGAGC |
| EZH2 ChIP-qPCR-forward | CTACCCTCAGGACAAACTCTGAAT |
| EZH2 ChIP-qPCR-reverse | TACGAGCACATGAACCTAACATGA |

**Supplementary Methods**

**Preparation of lentiviruses for short hairpin RNA (shRNA)-mediated knockdown (KD) and overexpression**

The shRNA sequences against *METTL3*, *SOX4*, *EZH2* and *UPF1* were designed and cloned into the pLKO-puro lentiviral vector. To generate lentiviral particles, the constructed shRNA-expressing plasmid was co-transfected with packaging plasmids pVSVg and psPAX2 into human embryonic kidney 293T cells using Lipofectamine 3000 (Invitrogen, USA). pGBM cells, U87MG and U251 GBM cells were infected with the obtained lentiviruses to KD the expression of *METTL3*. To increase KD efficiency, we transduced the cells with a combination of shRNA pools. The shRNA sequences are listed in **Supplementary Table 1.**

The full-length sequence of human *METTL3* was amplified using genomic DNA from 293T cells as a template and cloned into the pLVX-Tight-puro vector. An METTL3 catalytic mutant (AA395-398, DPPW to APPA) was generated using a Directed Mutagenesis Kit (NEB). Lentiviruses expressing Flag-METTL3 and rtTA were produced separately using the method described above. U87MG cells were infected with both lentiviruses and then screened using puromycin at a concentration of 3 μg/mL and G418 at a concentration of 100 μg/mL. Next, the expression of Flag-METTL3 was induced by adding 3 μg/mL doxycycline. The full-length sequence of human *EZH2* and was amplified and cloned into pLVX-CMV-puro vector.

**Real-time qPCR analysis**

We used TRIzol reagent (Invitrogen, USA) to purify the total RNA from the samples. The RNA was reverse-transcribed into first-strand cDNA using a HiScript II RT SuperMix for qPCR (Vazyme, China). The final quantitative real-time PCR reaction mix contained 5 μL 2 × ChamQ Universal SYBR qPCR Master Mix (Vazyme, China). The relative expression levels were detected and analyzed by QuantStudio 6 Flex Real-Time PCR Systems (Applied Biosystems, USA). The primer sequences used for RT-qPCR analysis were listed in **Supplementary Table 2**. The relative mRNA expression level was calculated using the 2^-ΔΔCt^ method.

**Immunohistochemistry (IHC)**

For IHC staining, the xenograft tissues and GBM surgical specimens were formalin-fixed, processed, and paraffin-embedded. Sections (6 µm) were generated for IHC analysis. These sections were deparaffinized and rehydrated through an alcohol series followed by antigen retrieval in 0.01 mol/L sodium citrate buffer (pH 6.0) at 121 °C for 3–5 min. Endogenous peroxidase activity and nonspecific binding sites were blocked using 3% H2O2 and 5% bovine serum albumin (BSA), respectively. The blocked sections were incubated overnight at 4 °C with primary antibodies against METTL3 (Proteintech, 15073-1-AP; 1:500) and EZH2 (Cell Signaling Technology, #5246; 1:200) in PBS with 0.1% Tween 20 and 1% BSA, followed by a 1 h incubation with secondary antibodies. The slides were stained with the DAB detection system (Thermo Scientific, USA) and mounted with Immuno-mount (Thermo Fisher Scientific). The scoring criterion was the average percentage of positively stained cells in 10 randomly selected visual fields.

**Monitor the tumor growth with a Xenogen IVIS Spectrum system**

To monitor the tumor growth using a Xenogen IVIS Spectrum system (Caliper Life Sciences), each mouse was injected intraperitoneally with 0.1 mL of the d-luciferin substrate. Mice were then anesthetized using 2% isoflurane and placed in the IVIS system approximately 2 min after the d-luciferin injection. The specific imaging parameters (field of view, acquisition time, and F-stop) were adjusted to optimize the signal (i.e., capture the largest signal possible without saturating the image). These parameters were adjusted per scan as each group of mice had constantly differing signals on any given imaging day. Scanning was continued until the peak signal was captured, which was between 3 and 12 min after the injection of d-luciferin.

The bioluminescence images were analyzed using a built-in software on the Xenogen IVIS system. Two-dimensional regions of interest (ROIs) were manually drawn (by authors E.S. Mittra or H. Fan-Minogue) over the head and used to measure the photon flux per area (p/s/cm2/sr). The ROI size was not fixed, but rather adjusted per animal to encompass the entire region of photon flux.

**Apoptosis analysis**

To determine the cell apoptosis induced by TMZ or *METTL3* KD, the cells were resuspended in binding buffer, then stained with Annexin V-FITC and PI. Measured the ratio of apoptotic cells (FITC^+^PI^-^) by the CytoFLEX S flow cytometer (Beckman Coulter, USA).

**Immunoblotting and immunoprecipitation**

For immunoblot analysis, cells were lysed in NP-40 buffer (10 mM Tris pH 7.4, 150 mM NaCl, 1% Triton X-100, 1 mM EDTA pH 8.0, 1 mM EGTA pH 8.0, 1 mM PMSF, and 0.5% NP-40) with protease inhibitor cocktail (Millipore, USA) at 4 °C for 1 h. The lysates were added to 5 × loading dye and then separated by electrophoresis and transferred for 120 minutes at 200 mA to PVDF membranes (Millipore, USA). After blocking with 5% skim milk in 1 ×TBST, the membranes were probed with antibodies against, METTL3 (Abcam, ab195352; 1:1000), SOX4 (Abcam, ab86809; 1:1000), KMT5A (Cell Signaling Technology, #2996; 1:1000), SMARCA2 (Cell Signaling Technology, #12760; 1:1000), ARID1A (Cell Signaling Technology, #12354; 1:1000), SUZ12 (Cell Signaling Technology, #3737; 1:1000), EZH2 (Cell Signaling Technology, #5246; 1:1000), β-ACTIN (Proteintech, #60008-1-Ig; 1:1000), Anti-rabbit IgG, HRP-linked Antibody (Cell Signaling Technology, #7074; 1:2000) and Anti-mouse IgG, HRP-linked Antibody (Cell Signaling Technology, #7076; 1:2000) were used as secondary antibody and signals were enhanced by chemiluminescence (Millipore, USA).

For immunoprecipitation, cells were lysed in Pierce IP Lysis Buffer (Thermo Scientific, USA) with protease inhibitor cocktail on ice for 30 min. Cell lysates were incubated with the anti-HA antibody at 4 °C overnight with gentle shaking. Protein A/G beads were added and incubated with cell lysates at 4°C for another 3 h. After four washes with Pierce IP Lysis Buffer, the precipitated proteins were boiled for 8 min with 2 × loading dye and analyzed by immunoblotting.

**ChIP-qPCR assay**

After different treatment, the cells were cross-linked with 1% (v/v) formaldehyde at room temperature for 10 min and then processed using the SimpleChIP Enzymatic Chromatin IP Kit (Cell Signaling Technology, USA) according to manufacturer’s instructions. The subsequent qPCR was conducted using the 2 × ChamQ Universal SYBR qPCR Master Mix (Vazyme, China). The primers used for ChIP-qPCR are listed in **Supplementary Table 2**.

**Dual-luciferase reporter assay**

The promoter region (3000 bp) of *METTL3* was amplified and cloned into the psiCheck2 plasmid (Promega). The HEK293T cells were seeded in a 12-well plate and transfected with the reporter plasmid. At 24 hours after transfection, the cells were infected with indicated shRNA lentiviruses. Following 72 h of transfection, The Dual-Glo® Luciferase Assay System (Promega Madison, WI) was used to measure reporter activity according to the manufacturer’s protocol. Renilla luciferase worked as the internal control to normalize results.

**DNA damage assay**

HCS DNA Damage Kit (Thermo Fisher Scientific, USA) was used to perform DNA damage assay. U87MG_TMZ_R cells were seeded in 96-well plates in 100 μL Gibco® Dulbecco's Modified Eagle's medium containing 10% FBS, at a density of 2 × 10^3^ cells per well with or without TMZ. The cells were incubated at 37 ℃ in a humidified 5% CO2 atmosphere. After TMZ treatment for 72 h, HCS DNA Damage Kit was used to detect the degree of DNA damage as the manufacturer’s protocol.

**RNA-seq transcriptome analysis**

RNA was extracted from METTL3-KD and control U87MG_TMZ_R cells using TRIzol solution (Life Technologies, USA). The integrity of the RNA was performed using the Agilent Bioanalyzer 2100. The paired-end reads were generated by the Illumina® Hi-Seq 2500 platform and mapped to the human genome. DESeq, was applied for transcription quantification and differential expression analysis using a cutoff of P<0.05. Changes in splicing isoforms were analyzed by replicate multivariate analysis of transcript splicing (rMATS), a Bayesian statistical framework.

**Gene Set Enrichment Analysis (GSEA)**

GSEA was carried out by the Broad Institute website (<http://www.broadinstitute.org/gsea/index.jsp> ). All GSEA analyses in this study were performed via the Java GSEA implementation. The input of GSEA is a gene expression matrix, in which the samples are divided into 2 groups (A and B). Firstly, all genes are ranked according to the FoldChange value (such as A-VS-B), and the top of the ranked gene list can be regarded as up-regulated differential genes (relatively enriched in A). Genes at the bottom are down-regulated differential genes (relatively enriched in B). The position of genes in a predefined gene set is then assessed in the gene list above, and whether the genes in this gene set are relatively concentrated in group A or B are calculated. Therefore, x-axis in Figure 1E means RANK in ordered dataset，and black-lines represent the sorting position of each gene in the gene set.

**Gene ontology (GO) analysis**

Biological functions were analyzed using DAVID (<http://david.abcc.ncifcrf.gov/>). GO terms with p < 0.05 were determined to be statistically significant. Bubble Plots were constructed using R Studio 1.2 with R packages ggplot2.

**Identification and clustering of enriched motifs**

The m^6^A motifs were detected in an in-house method using all identified peaks. Motifs were identified by counting the occurrence of 6 nucleotide k-mers in the m^6^A-IP and control group. Analysis was limited by p < 0.05.
